# IFN-γ extends the immune functions of Guanylate Binding Proteins to inflammasome-independent antibacterial activities during *Francisella novicida* infection

**DOI:** 10.1371/journal.ppat.1006630

**Published:** 2017-10-02

**Authors:** Pierre Wallet, Sacha Benaoudia, Amandine Mosnier, Brice Lagrange, Amandine Martin, Helena Lindgren, Igor Golovliov, Fanny Michal, Pauline Basso, Sophia Djebali, Angelina Provost, Omran Allatif, Etienne Meunier, Petr Broz, Masahiro Yamamoto, Bénédicte F. Py, Eric Faudry, Anders Sjöstedt, Thomas Henry

**Affiliations:** 1 CIRI, Centre International de Recherche en Infectiologie, Inserm, U1111, Université Claude Bernard Lyon 1, CNRS, UMR5308, École Normale Supérieure de Lyon, Univ Lyon, Lyon, France; 2 Laboratory for Molecular Infection Medicine Sweden and Department of Clinical Microbiology, Umeå University, Umeå, Sweden; 3 University of Grenoble Alpes, CNRS, ERL5261, CEA, BIG-BCI, Inserm, U1036, Grenoble, France; 4 Institut de Pharmacologie et de Biologie Structurale (IPBS), CNRS, Université Paul Sabatier (UPS), Toulouse, France; 5 Focal Area Infection Biology, Biozentrum, University of Basel, Basel, Switzerland; 6 Department of Immunoparasitology, Research Institute for Microbial Diseases, Osaka University, Osaka, Japan; Stanford University School of Medicine, UNITED STATES

## Abstract

Guanylate binding proteins (GBPs) are interferon-inducible proteins involved in the cell-intrinsic immunity against numerous intracellular pathogens. The molecular mechanisms underlying the potent antibacterial activity of GBPs are still unclear. GBPs have been functionally linked to the NLRP3, the AIM2 and the caspase-11 inflammasomes. Two opposing models are currently proposed to explain the GBPs-inflammasome link: i) GBPs would target intracellular bacteria or bacteria-containing vacuoles to increase cytosolic PAMPs release ii) GBPs would directly facilitate inflammasome complex assembly. Using *Francisella novicida* infection, we investigated the functional interactions between GBPs and the inflammasome. GBPs, induced in a type I IFN-dependent manner, are required for the *F*. *novicida*-mediated AIM2-inflammasome pathway. Here, we demonstrate that GBPs action is not restricted to the AIM2 inflammasome, but controls in a hierarchical manner the activation of different inflammasomes complexes and apoptotic caspases. IFN-γ induces a quantitative switch in GBPs levels and redirects pyroptotic and apoptotic pathways under the control of GBPs. Furthermore, upon IFN-γ priming, *F*. *novicida*-infected macrophages restrict cytosolic bacterial replication in a GBP-dependent and inflammasome-independent manner. Finally, in a mouse model of tularemia, we demonstrate that the inflammasome and the GBPs are two key immune pathways functioning largely independently to control *F*. *novicida* infection. Altogether, our results indicate that GBPs are the master effectors of IFN-γ-mediated responses against *F*. *novicida* to control antibacterial immune responses in inflammasome-dependent and independent manners.

## Author summary

The cell-intrinsic immunity is defined as the mechanisms allowing a host cell infected by an intracellular pathogen to mount effective immune mechanisms to detect and eliminate pathogens without any help from other immune cells. In infected macrophages, the Guanylate Binding Proteins (GBPs) are immune proteins, induced at low levels in a cell autonomous manner by endogenous type I IFN or at high levels following IFN-γ production by innate and adaptive lymphocytes. The antibacterial activity of GBPs has been recently tightly linked to the inflammasomes. Inflammasomes are innate immune complexes leading to inflammatory caspases activation and death of the infected cell. *Francisella novicida*, a bacterium replicating in the macrophage cytosol, is closely related to *F*. *tularensis*, the agent of tularemia and is used as a model to study cytosolic immunity. GBPs contribute to *F*. *novicida* lysis within the host cytosol leading to DNA release and AIM2 inflammasome activation. In addition to their regulation of the AIM2 inflammasome, we identified that GBPs also control several other pyroptotic and apoptotic pathways activated in a hierarchical manner. Furthermore, we demonstrate that IFN-γ priming extends GBPs anti-microbial responses from the inflammasome-dependent control of cell death to an inflammasome-independent control of cytosolic bacterial replication. Our results, validated in a mouse model of tularemia, thus segregate the antimicrobial activities of inflammasomes and GBPs as well as highlight GBPs as the master effectors of IFN-γ-mediated cytosolic immunity.

## Introduction

Intracellular pathogens have evolved sophisticated mechanisms to invade and replicate within host cells. In parallel, multi-cellular organisms have evolved multiple mechanisms allowing a host cell to detect microbial infection and to mount an effective antimicrobial response. Key actors of the host cell intrinsic immunity include the Guanylate Binding Proteins (GBPs)[1–3]. GBPs constitute a family of interferon-inducible dynamin-like GTPases [4,5]. 11 GBPs are encoded by the murine genome in two clusters on chromosomes 3 and 5 [6,7]. The antimicrobial functions of GBPs are still poorly understood. One key mechanism of GBPs' potent antimicrobial activity resides in their ability to target and disrupt pathogen-containing vacuoles. Indeed, chromosome 3-encoded GBPs (*Gbp*^chr3^) are required to disrupt *Toxoplasma gondii* parasitophorous membrane and to control the parasite replication in mice [8,9]. Similarly, *Gbp*^chr3^ are required to lyse the *Salmonella*-containing vacuole leading to the release of this bacterium into the host cytosol and to the subsequent activation of the caspase-11 non-canonical inflammasome [10].

Cooperation between GBPs and the NLRP3, the AIM2 and the non-canonical caspase-11 inflammasome complexes have emerged recently as central to the innate immune responses against intracellular bacteria [10–14]. However, the functional molecular links between GBPs and the inflammasomes remain unclear. GBP5 was described to bind NLRP3 and directly promote NLRP3-dependent inflammasome assembly [14]. This finding was later challenged by several groups [10,15]. Chromosome 3-encoded GBPs are key host factors to trigger the non-canonical caspase-11 inflammasome in macrophages infected with various Gram-negative bacteria [16] including *Salmonella typhimurium* [10], *Legionella pneumophila* [12] and *Chlamydia trachomatis* [11]. Three different models have been proposed to explain the role of GBPs in promoting non-canonical inflammasome activation. Similarly to what have been demonstrated for IFN-γ-inducible Immunity-Related GTPases (IRG) in their antimicrobial role against *Toxoplasma gondii* [17], GBPs might disrupt the *Salmonella*-containing vacuole leading to the release of this bacterium and its associated LPS into the host cytosol [10]. Alternatively, GBPs might orchestrate the recruitment of IRGB10 onto cytosolic bacteria to liberate LPS for sensing by caspase-11 [16]. Finally, as GBPs can promote caspase-11 activation without any detectable recruitment around *Chlamydia muridarum* inclusions and upon *Legionella* LPS transfection into the host cytosol, Coers and colleagues suggested that GBPs might directly facilitate caspase-11 activation [11,12].

The link between GBPs and the AIM2 inflammasome is less controversial and has been mostly studied by our group and others in macrophages infected with *Francisella novicida* [13,15,18]. *F*. *novicida* is a close relative of *F*. *tularensis*, the agent of tularemia. The virulence of *Francisella* strains is linked to their ability to rapidly lyse the phagosome, escape into the host cytosol [19,20] and replicate within this compartment. This process is dependent on a cluster of genes in the *Francisella*-pathogenicity island [21,22], which encodes an atypical type VI secretion system [23]. GBP2 and GBP5 are recruited onto cytosolic *F*. *novicida* and are required to lyse bacteria and release the bacterial genomic DNA into the host cytosol. DNA in the host cytosol is then recognized by AIM2 [13,15,24–26]. Recently, GBPs were demonstrated to be required for IRGB10 recruitment onto cytosolic *F*. *novicida* to mediate cytosolic bacterial killing, DNA release and AIM2 inflammasome activation [16].

Chromosome 3-encoded GBPs and the AIM2 inflammasome are both equally required to resist *F*. *novicida* infection *in vivo* [13,15]. Yet, whether GBPs might have anti-*F*. *novicida* functions dependent on other inflammasomes or inflammasome-independent antibacterial responses remain unclear [10,16]. In this work, we demonstrate that GBPs control *F*. *novicida*-mediated host cell death in a hierarchical manner implicating at least 3 different canonical and non-canonical inflammasome complexes as well as apoptotic caspases 8, 9 and 3. Furthermore, we demonstrate that upon IFN-γ treatment, GBPs control *F*. *novicida* replication independently of the canonical and non-canonical inflammasome pathways and independently of macrophage cell death. IFN-γ-, GBPs-mediated inhibition of intracellular bacterial growth was also effective during *F*. *tularensis* spp. *holarctica* Live Vaccine Strain infection, while IFN-γ was inefficient to block the replication of the highly virulent *F*. *tularensis* SCHU S4 strain. Finally, we demonstrate *in vivo* that IFN-γ-mediated host protection against *F*. *novicida* is largely GBPs-dependent and inflammasome-independent. Our work thus positions the GBPs as the master effectors of the IFN-γ-mediated anti-*F*. *novicida* responses.

## Results

### *Gbp*^chr3^ and the inflammasome control in vivo host resistance to *F*. *novicida* in a non-redundant manner

**Fig 1:**
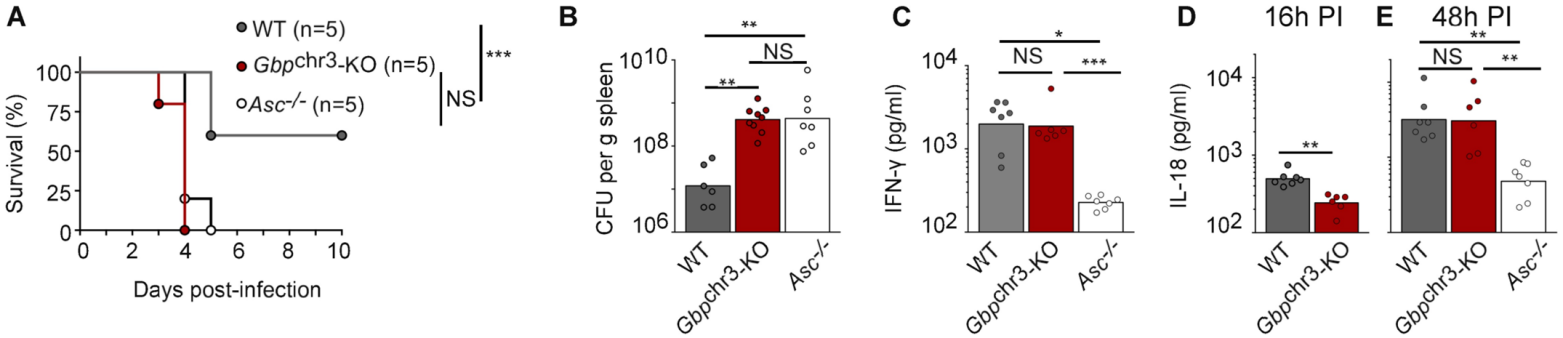
*Gbp*^chr3^ and the inflammasome control the *in vivo* host resistance to *F*. *novicida* in a divergent manner. Mice of the indicated genotypes were subcutaneously (sc) injected with 5×10^3^ (A-B) or 4×10^5^ (C-E) *F*. *novicida*. (A) Survival was monitored twice a day for 10 days. (B) Bacterial burden in the spleen, (C) IFN-γ or (D, E) IL-18 levels in the serum were quantified at 16 h (D) and 48 h post-inoculation (C, E). IFN-γ concentration at 16 h post-inoculation was under the limit of detection. Each symbol represents the value of an individual mouse; the bars indicate the geometric mean. (A-E) one experiment representative of one (D), to at least three (A-C, E) independent experiments is shown. Mantel Cox log-rank test (A), Kruskal-Wallis analysis with Dunn's correction for multiple comparisons (B, C, E) and Mann-Whitney test (D) were performed.

We and others have previously reported that *Gbp*^chr3^-deficient and inflammasome-deficient mice (*Aim2*^*-/-*^, *Asc*^*-/-*^ and *Casp1/Casp11*^*-/-*^ mice) were highly susceptible to *F*. *novicida* infection [13,15,24,25,27,28]. *In vitro* studies have demonstrated that GBPs act upstream of the AIM2 inflammasome [13,15,16] suggesting that the *Gbp*^chr3^ deficiency should phenocopy deficiencies in the AIM2 inflammasome. In a survival experiment, *Gbp*^chr3^-KO and *Asc*^*-/-*^ were almost as susceptible to *F*. *novicida* infection (Fig 1A) although we consistently noticed that *Gbp*^chr3^KO mice died slightly faster than *Asc*^-/-^ mice. Similarly, *Gbp*^chr3^-KO and *Asc*^*-/-*^ mice displayed very high bacterial burden both in the spleen and in the liver at day 2 post-infection (PI) with an average of 40-fold more bacteria than WT mice (Fig 1B). Surprisingly, at 48 h PI, IFN-γ level in the serum of *Gbp*^chr3^-deficient mice reached WT levels while in agreement with previous work [28], IFN-γ level in the serum of *Asc*^*-/-*^ mice was strongly decreased (Fig 1C). We and others have previously reported that early (16 h PI [13], 24 h PI [16]) IL-18 production *in vivo* is GBPs-dependent, a finding which we reproduced here (Fig 1D). However, at later time points (48 h PI), IL-18 levels were similar in *Gbp*^chr3^-KO and in WT mice (Fig 1E). As expected, IL-18 levels in infected *Asc*^*-/-*^ mice were not statistically different from the levels observed in uninfected mice. These results indicated that inflammasome activation was only delayed *in vivo* in *Gbp*^chr3^-deficient mice. The increase in IL-18 levels observed over 48h in infected *Gbp*^chr3^-KO mice likely explained the high IFN-γ serum level observed in these mice. The high susceptibility of *Gbp*^chr3^-KO mice to *F*. *novicida* infection despite high levels of circulating IFN-γ is remarkable since IFN-γ is considered to be one of the most important cytokine to fight *Francisella* infection [29–31]. This conundrum led us to hypothesize that GBPs might be the main IFN-γ effector in *F*. *novicida*-infected mice. Furthermore, this result indicates that while the overall susceptibility of *Gbp*^chr3^-deficient mice and *Asc*^*-/-*^ mice to *F*. *novicida* infection are similar, the *in vivo* antibacterial mechanisms of GBPs are, at least partially, independent of the inflammasome.

### IFN-γ priming induces a quantitative shift in GBPs levels

The action of GBPs against *F*. *novicida* has been mostly studied in unprimed macrophages [13,15,16]. Under these conditions, GBPs induction relies on the endogenous recognition of nucleic acids by the cGAS pathway, secretion of type I IFN, signaling through the type I IFN receptor (IFNAR1) and activation of the IRF-1 pathway [15,24,32]. Importantly, there was a drastic quantitative shift in GBP2 and GBP5 transcripts levels upon priming with IFN-γ compared with *F*. *novicida*-mediated endogenous induction or to induction following IFN-β priming (S1A Fig). Indeed while GBP2 transcript levels increased by a factor of 15 upon *F*. *novicida* infection, IFN-γ priming of infected macrophages, led to a 325-fold increase in GBP2 transcript levels relative to its level in uninfected macrophages. As previously reported, this very strong induction likely results from synergistic NF-κB and IFN signaling [14,33,34]. Indeed, we obtained comparable levels of GBP2 induction when BMDMs were primed with both IFN-γ and Pam_3_CSK_4_, a TLR2 agonist (S1A Fig). ProIL-1β transcript levels were not impacted by IFN-γ priming (S1C Fig) indicating that this synergy was specific for GBPs induction. Similar results were obtained while investigating GBP5 transcript levels (S1B Fig) or while monitoring GBP2 and GBP5 protein levels (S1D Fig). This quantitative shift in GBPs levels upon IFN-γ treatment led us to investigate the impact of IFN-γ on the *F*. *novicida*-mediated cell death.

### GBPs control *F*. *novicida*-mediated cell death in an AIM2 inflammasome-dependent and -independent manner

**Fig 2:**
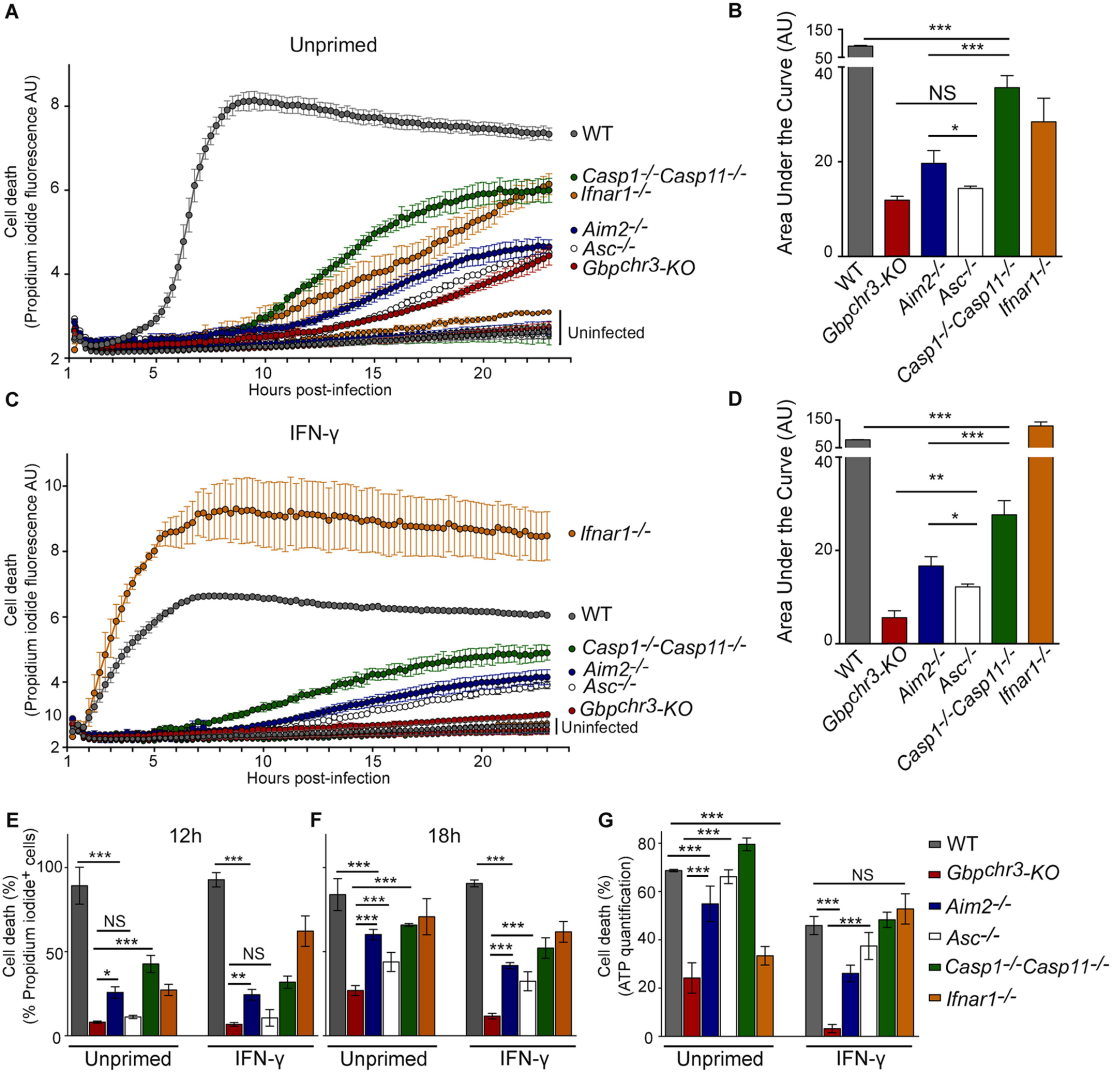
IFN-γ-induced GBPs control *F*. *novicida*-mediated cell death in an inflammasome-dependent and -independent manner. Cell death was measured in real-time by assessing propidium iodide fluorescence in (A, B) unprimed or (C, D) IFN-γ-primed (100 U/ml for 16 h) BMDMs from the indicated genotypes infected or not with *F*. *novicida* at a MOI of 10. (B, D) Kinetics were quantified by calculating the corresponding area under the curve. (E-F) The number of propidium iodide positive cells was quantified from time-lapse video-microscopy images (see S3 Fig and S1 and S2 Movies) at 12 h (E) and 18 h (F) post-infection. (G) ATP-based cell viability was monitored at 24 h post-infection. Cell death is shown. (A-G) Mean and s.d. are shown. One experiment representative of two (E, F) to at least three independent experiments is shown. (B, D, E-G) One-way ANOVA analysis was performed with Tukey's correction for multiple comparisons.

In *F*. *novicida*-infected bone marrow-derived macrophages (BMDMs), the only reported cell death pathways are dependent on the AIM2/ASC complex [24,25,27,35]. By monitoring, in real time, propidium iodide influx and fluorescence over 24 h in unprimed macrophages deficient for various inflammasome components, we observed substantial differences in the kinetics of propidium iodide between various knock-out macrophages suggesting that several cell death pathways were engaged following *F*. *novicida* infection (Fig 2A). These differences in the kinetics of propidium iodide incorporation/ fluorescence were demonstrated to be statistically significant by calculating the area under the curve corresponding to each kinetics (Fig 2B). As previously described [13,15,24,25], in the absence of IFN-γ priming, *F*. *novicida*-infected BMDMs died in an AIM2-dependent manner. Indeed, at MOI 10, propidium iodide incorporation/fluorescence sharply increased around 6 h post-infection in WT macrophages while *Aim2*^*-/-*^ BMDMs presented cell death kinetics delayed by more than 7 h compared with that of WT macrophages. Interestingly, incorporation/fluorescence increase of propidium iodide was significantly further delayed in *Gbp*^chr3^-KO BMDMs compared to *Aim2*^*-/-*^ BMDMs suggesting that GBPs control both AIM2-dependent and -independent cell death pathways. Accordingly, by monitoring macrophage cell death in real time and in single cells using time-lapse microscopy (Fig 2E and 2F; S3 Fig), we clearly observed that the number of propidium iodide-positive cells increased significantly later in *Gbp*^chr3^-KO BMDMs compared to *Aim2*^*-/-*^ BMDMs. This difference in cell death kinetics was exacerbated in the presence of IFN-γ priming (Fig 2C, 2D and 2F). Finally, to exclude any bias associated with propidium iodide incorporation, we used a luminescent cell viability assay based on the quantitation of ATP, a signature of metabolically active cells (CellTiter-Glo; Fig 2G). This assay confirmed that *Gbp*^chr3^-KO BMDMs survived longer than *Aim2*^*-/-*^ BMDMs upon *F*. *novicida* infection. Altogether, our data strongly suggest that GBPs control both AIM2-dependent and -independent cell death/survival pathways.

### *F*. *novicida* infection triggers GBPs-dependent AIM2-, NLRP3-dependent canonical and non-canonical caspase-11 inflammasomes

**Fig 3:**
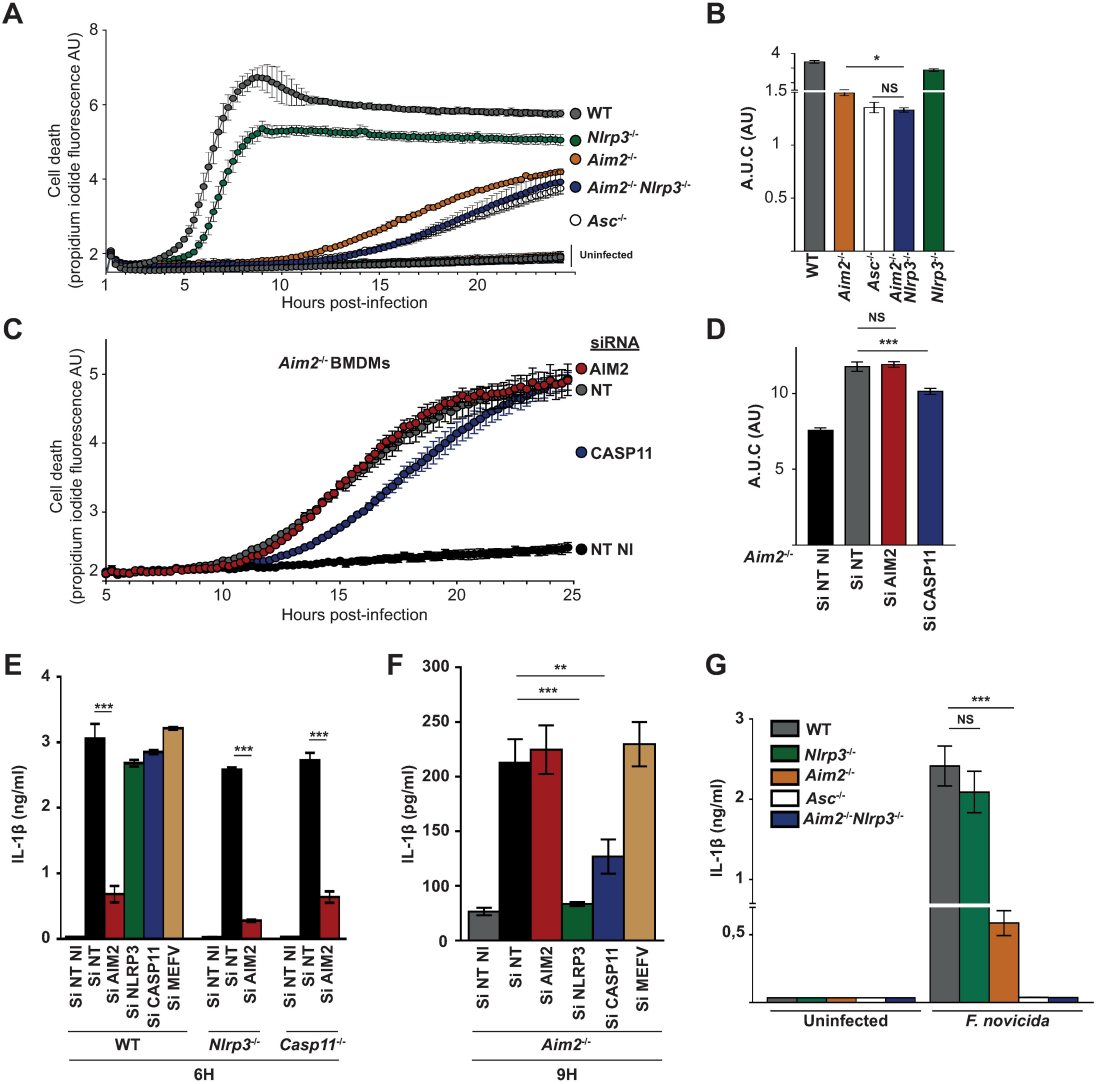
*F*. *novicida* infection triggers AIM2-, NLRP3-dependent canonical and caspase-11 non-canonical inflammasomes activation in a hierarchical manner. (A-D) Cell death was measured in real-time by assessing propidium iodide fluorescence in BMDMs of the indicated genotype infected with *F*. *novicida* at an MOI of 10. (B, D) Kinetics were quantified by calculating the corresponding area under the curve. (C) *Aim2*^-/-^ BMDMs were treated with Non-Targeting (NT) or indicated gene-specific siRNA. (E-G) IL-1β levels in the supernatant of BMDMs of the indicated genotype treated (E, F) or not (G) with the indicated siRNA and infected with *F*. *novicida* at an MOI of 10 were quantified at 6 or 9 h post-infection as indicated. Graphs show mean and s.d. of triplicate assays. Data are representative of three independent experiments. (B, D, E-G) One-way ANOVA analysis was performed with Tukey's correction for multiple comparisons. (NI: Not infected).

In *F*. *novicida*-infected murine macrophages, the only inflammasomes described so far are dependent on AIM2, while our results (Fig 2A–2G) clearly demonstrate that *F*. *novicida*-mediated cell death can proceed independently of AIM2. We thus used *Aim2*^*-/-*^ macrophages to unravel other cell death pathways controlled by GBPs. Using siRNA, we first knock-downed various inflammasome NLRs (S4A Fig) and observed a contribution of NLRP3 in *F*. *novicida*-mediated cell death (S4B Fig). To confirm this result, we generated *Aim2*^*-/-*^*/Nlrp3*^*-/-*^ mice and compared their BMDMs response with that of BMDMs single knock-out for *Aim2* (Fig 3A and 3B). *Aim2*^*-/-*^*/Nlrp3*^*-/-*^ BMDMs displayed a kinetics of propidium iodide incorporation/fluorescence slower than that of *Aim2*^*-/-*^ BMDMs, indicating that in the absence of AIM2, NLRP3 controls *F*. *novicida*-mediated cell death. *Aim2*^*-/-*^*/Nlrp3*^*-/-*^ BMDMs phenocopied *Asc*^*-/-*^ BMDMs indicating that activation of the canonical inflammasome pathways in *F*. *novicida*-infected BMDMs is exclusively dependent on AIM2 and NLRP3.

To assess whether the non-canonical caspase-11 inflammasome could be involved in the AIM2-independent detection of *F*. *novicida*, we used siRNA against caspase-11. We observed a consistent and significant delay in *Aim2*^*-/-*^ macrophage death upon treatment with a caspase-11 siRNA (Fig 3C and 3D and S4B Fig) indicating that while *F*. *novicida* is largely able to escape caspase-11 detection ([36], Fig 3E, S4C and S4D Fig), caspase-11 may contribute to macrophage cell death at late time points of the infection in the absence of AIM2. The contribution of caspase-11 in the immune response of *Aim2*^*-/-*^ macrophages was further strengthened by investigating IL-1β release. Indeed, *caspase-11* expression knock-down strongly decreased the late secretion of IL-1β observed in *Aim2*^*-/-*^ macrophages (Fig 3F). *Nlrp3* expression knock-down or knock-out also consistently decreased IL-1β release in *Aim2*^*-/-*^ macrophages (Fig 3F and 3G). This decrease may be partly due to its involvement downstream of caspase-11 [37].

Altogether, our data demonstrate the involvement of at least two sequential cell death pathways (mediated by the sensors AIM2, NLRP3 and caspase-11) elicited in response to *F*. *novicida* infection. The extensive survival of *Gbp*^chr3^-KO BMDMs strongly suggests that GBPs contribute to activation of these three inflammasome complexes during *F*. *novicida* infection. Of note, at 24 h post-infection in absence of IFN-γ, propidium iodide incorporation/ fluorescence was similar in *Asc*^*-/-*^ and *Gbp*^chr3^-KO BMDMs. The late cell death occurring in *Gbp*^chr3^-KO BMDMs was associated with IL-1β release while as expected no IL-1β was observed in the supernatant of *Asc*^*-/-*^ BMDMs (S5 Fig). This result suggests that while chromosome 3-encoded GBPs are instrumental in promoting fast inflammasome activation upon *F*. *novicida* infection, they are not strictly required to trigger inflammasome activation likely explaining the bi-phasic dependence of IL-18 serum level on GBPs (Fig 1D and 1E). Interestingly, analysis of macrophages deficient for both *Asc* and *Gbp*^chr3^ demonstrated a strong delay in propidium iodide incorporation/fluorescence increase compared to that of *Asc*^*-/-*^ and *Gbp*^chr3^-KO BMDMs (S6A and S6C Fig) providing genetic evidence that GBPs can act independently of the canonical inflammasomes.

### GBPs control *F*. *novicida*-mediated apoptotic pathways

**Fig 4:**
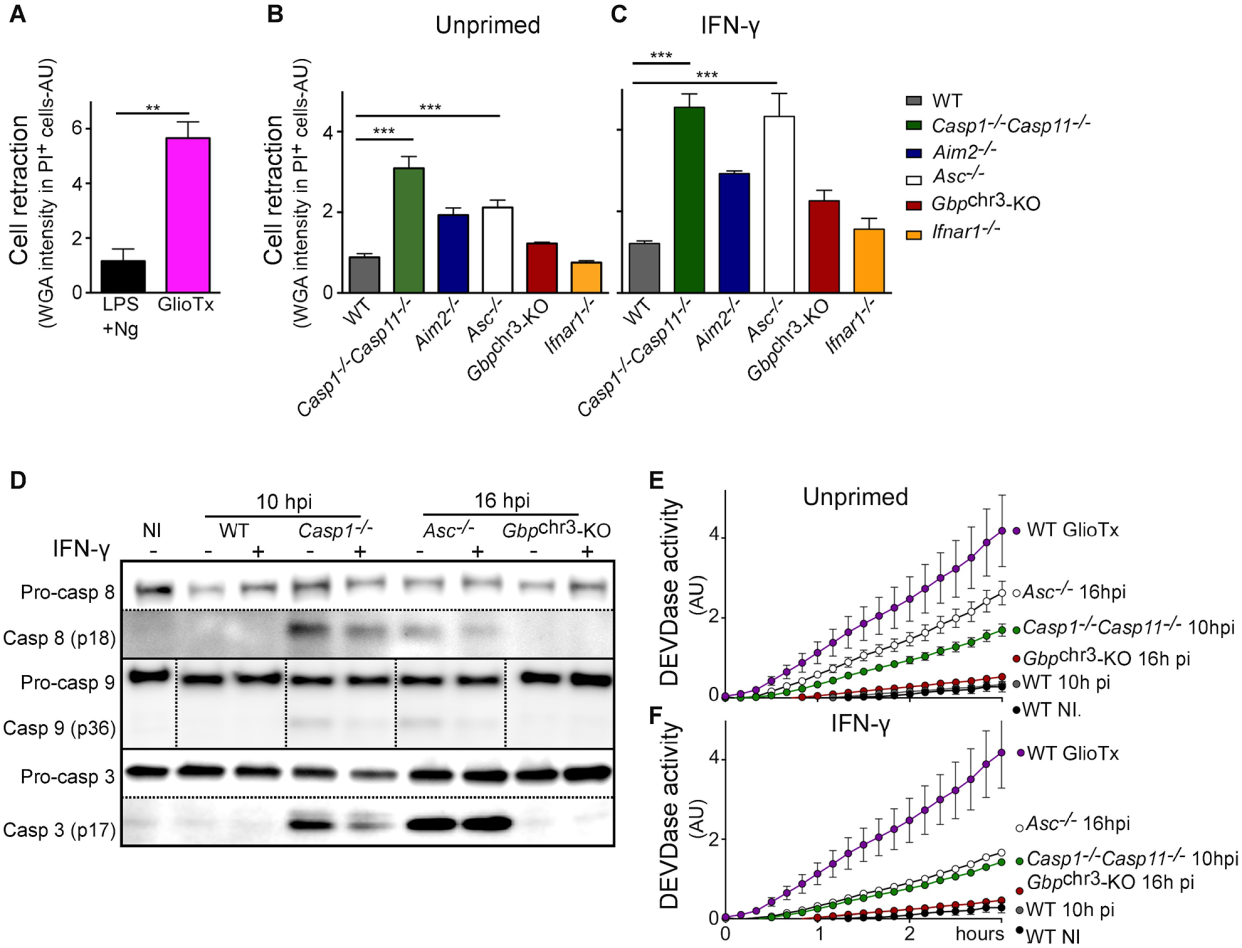
GBPs control apoptosis in inflammasome-deficient BMDMs. (A-C) Cell retraction in propidium iodide positive cells was assessed by quantifying wheat germ agglutinin staining intensity at (A) 90 minutes post Nigericin (Ng, a pyroptotis stimulus) or post-Gliotoxin (GlioTx, an apoptotic stimulus) or at (B, C) 21 h post-infection in (A, B) unprimed or (C) IFN-γ-primed (100 U/ml for 16 h) BMDMs from the indicated genotypes infected (B, C) or not with *F*. *novicida* at a MOI of 10. (D) Lysates from BMDMs from the indicated genotypes infected or not with *F*. *novicida* at a MOI of 10 were analyzed by Western blotting analysis. IFN-γ-priming (100 U/ml for 16 h) is indicated. The dotted vertical lines in Casp9 Western blot illustrate that the samples from a single original Western blot gel/ image were reorganized to fit the indicated order without any other image manipulation. The dotted horizontal lines in Casp8 and Casp3 Western blot indicate images from two different exposure times or from the use of two different primary antibodies (pro- and cleaved Casp3), respectively. (E, F) DEVDase activity was analyzed using a fluorogenic caspase3/7 substrate at 4 h post-GlioTx addition or at the indicated time post-infection in BMDMs from the indicated genotypes infected or not with *F*. *novicida* at a MOI of 10 and primed (F) or not (E) with IFN-γ (100 U/ml for 16 h). (A-C, E) Mean and s.d. are shown. (A-F) One experiment representative of two independent experiments is shown. Unpaired t-test (A) and one-way ANOVA analysis with Tukey's correction for multiple comparisons (B-C) were performed. (NI: non-infected).

In addition to uncovering AIM2-independent cell death pathways, real time cell death analysis of *F*. *novicida*-infected BMDMs (Figs 2A–2F and 3A–3D and S3 and S4 Figs) revealed both known and unsuspected cell death pathways. In agreement with previously reported LDH-release quantifications [13], IFN-γ could complement *Ifnar1*^*-/-*^ BMDMs inability to rapidly undergo cell death upon *F*. *novicida* infection (Fig 2). Furthermore, as previously described [35,38], *Casp1/Casp11*^*-/-*^ BMDMs died significantly faster than *Aim2*^*-/-*^ BMDMs (Fig 2A–2G, S3 Fig). Apoptosis is associated with cell retraction while pyroptosis is associated with cell swelling [39]. We took advantage of wheat germ agglutinin (which binds cell membrane glycoproteins) staining intensity to quantify cell retraction and of well-characterized stimuli triggering pyroptosis (LPS + nigericin [40]) and apoptosis (gliotoxin [41]) to validate this cell retraction quantification (Fig 4A). *F*. *novicida*-infected *Casp1/Casp11*^*-/-*^ BMDMs cell death proceeded via a major cell retraction (S1 Movie, Fig 4B and 4C), a morphological feature of apoptosis. The ability of AIM2/ASC complex to recruit caspase-8 and trigger apoptosis in *Casp1/Casp11*^*-/-*^ BMDMs [35,38] may explain the kinetics of cell death observed in *Casp1/Casp11*^*-/-*^ BMDMs. Indeed, at 10 h post-infection, we observed processing of the apoptotic caspases-8, 9 and 3 in infected *Casp1/Casp11*^*-/-*^ BMDMs. As expected and as previously reported [35], we did not observe such apoptotic caspases processing in WT pyroptotic macrophages (Fig 4D). Furthermore, a strong DEVDase activity suggestive of active caspase-3 (or caspase-7) was observed in *Casp1/Casp11*^*-/-*^ BMDMs but not in WT macrophages (Fig 4E and 4F). Interestingly, the kinetics of propidium iodide incorporation/fluorescence in infected *Gbp*^chr3^-KO BMDMs were even slower than the corresponding kinetics of *Casp1/Casp11*^*-/-*^ BMDMs (Fig 2A–2D). This difference strongly suggests that GBPs not only control pyroptosis, but also has the ability to control apoptotic pathways.

This conclusion was further strengthened by comparing cell death kinetics, morphological and molecular features of IFN-γ-primed *Asc*^*-/-*^ and *Gbp*^chr3^-KO BMDMs. Priming with IFN-γ accelerated the cell death kinetics in the different macrophages with one notable exception (Fig 2C–2F). Indeed, we did not detect any substantial propidium iodide incorporation/fluorescence increase in IFN-γ-primed *Gbp*^chr3^-KO macrophages while IFN-γ-primed *Asc*^*-/-*^ BMDMs died although with delayed kinetics compared with that of WT macrophages (Fig 2C–2F, S3B Fig). The death of *Asc*^-/-^ BMDMs was confirmed by addition of triton (TX100) at the end of the 24 h kinetics. Indeed, TX100 treatment did not further increase propidium iodide incorporation/fluorescence in *Asc*^-/-^ BMDMs (S2B Fig). In contrast, TX100 addition in *Gbp*^chr3^-KO BMDMs led to a strong increase in propidium iodide fluorescence indicating that the plasma membrane of most *Gbp*^chr3^-KO BMDMs was still intact (and not permeable to propidium iodide) at 24 h PI. The relatively lower intensity of propidium iodide fluorescence in *Asc*^-/-^ BMDMs compared to WT BMDMs might be related to intrinsic differences in propidium iodide incorporation/fluorescence upon incorporation in apoptotic vs. pyroptotic nuclei. Cell death of IFN-γ-primed *Asc*^*-/-*^ BMDMs was associated with cell retraction (S2 Movie and Fig 4C) and was reminiscent of the morphological cell death observed in *Casp1/Casp11*^*-/-*^ BMDMs although it progressed with a ≈6 h delay compared to the latter BMDMs. Accordingly, apoptotic caspase-8/9/3 processing and DEVDase activity were clearly detectable in *Asc*^*-/-*^ BMDMs infected for 16 h corroborating the morphological features and demonstrating that at late time points of infection, *Asc*^*-/-*^ BMDMs die by apoptosis.

The extensive survival of *Gbp*^chr3^-KO BMDMs upon IFN-γ priming was confirmed using CellTiter-Glo assay (Fig 2G). Furthermore, we observed a limited number of cell death events in infected *Gbp*^chr3^-KO BMDMs using time-lapse microscopy (Fig 2F, S3 Fig and S2 Movie) suggesting that a limited GBPs-independent cell death pathway occurs at late time points of infection. The cell survival effect of IFN-γ on *Gbp*^chr3^-KO BMDMs (compare Figs 2A and 2B with 2C and 2D; S3A with S3B and S6A with S6B, 2F and 2G, S3C and S6C) correlates with a decrease in IL-1β release (S5 Fig). This result suggests that in infected *Gbp*^chr3^-KO BMDMs, IFN-γ might inhibit inflammasome activation possibly via nitrosylation, an IFN-inducible mechanism previously reported to inhibit inflammasome activation [42,43]. While the three different cell death/survival assays showed subtle differences [44], these assays converge to indicate that upon IFN-γ priming, *Gbp*^chr3^-KO BMDMs survive better than *Casp1/Casp11*^*-/-*^ and *Asc*^*-/-*^ BMDMs suggesting that GBPs control both pyroptotic and apoptotic pathways. Importantly, the GBPs-mediated control of apoptosis was confirmed by generating mice doubly deficient for *Asc* and *Gbp*^chr3^. Macrophages deficient for both *Asc* and *Gbp*^chr3^ failed to demonstrate caspase-8/9 and 3 maturation and DEVDase activity in contrast to *Asc*^*-/-*^ macrophages (S6D and S6E Fig). Altogether, our results demonstrate that GBPs act as major cell death regulators by controlling numerous pyroptotic and apoptotic cell death pathways.

### IFN-γ-induced GBPs control bacterial killing independently of inflammasomes

**Fig 5:**
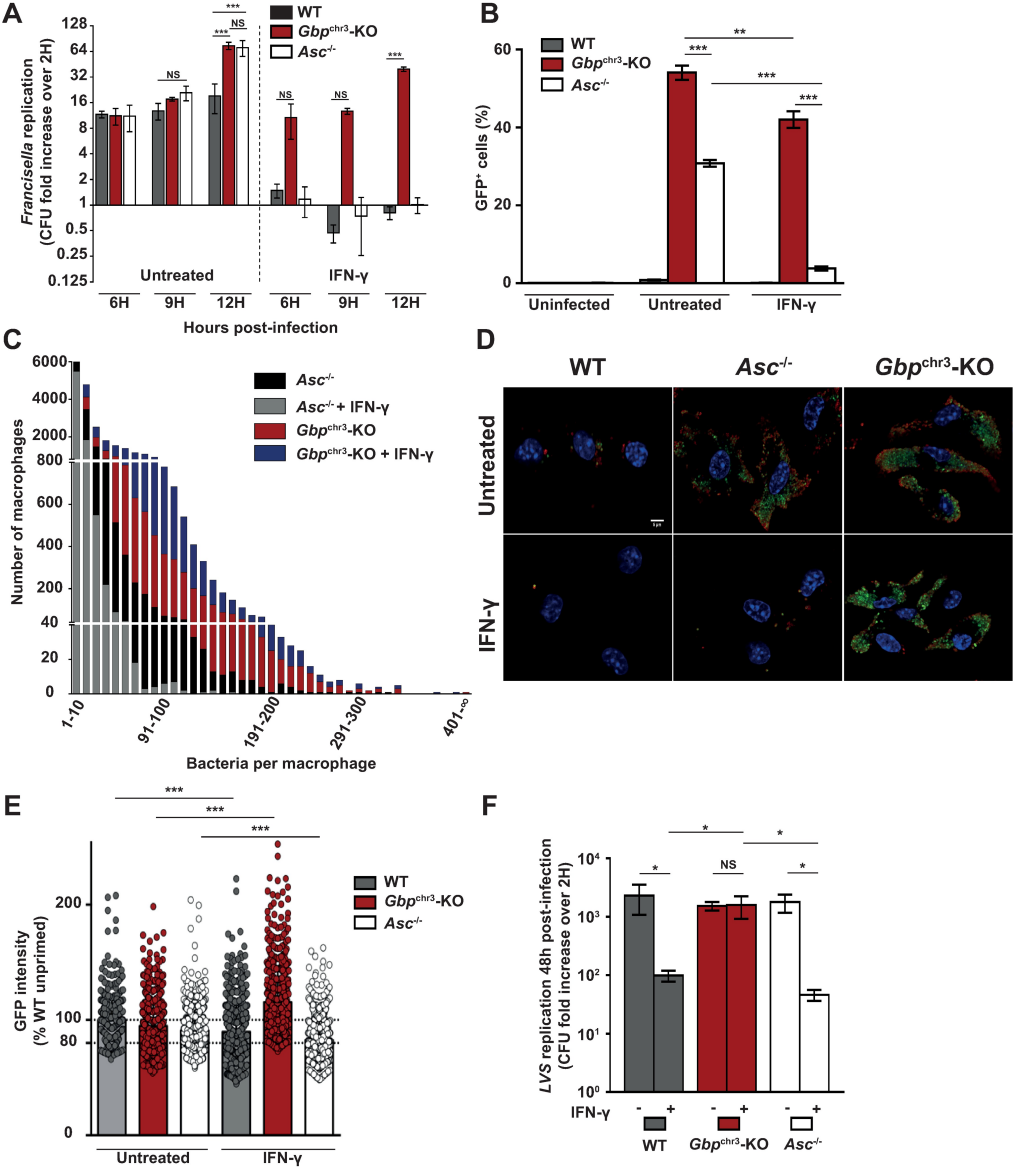
IFN-γ-induced GBPs control intracellular bacterial replication independently of inflammasomes. (A-F) BMDMs from the indicated genotypes were primed or not overnight with 100 U/ml of IFN-γ. BMDMs were infected with (A-E) *F*. *novicida* or (F) *F*. *tularensis* LVS at a multiplicity of infection (MOI) of 1 and 0.4, respectively. Intracellular bacterial burden was assessed by determination of viable counts at the indicated times post-infection. Results were normalized with viable counts detected at 2h post-infection. The corresponding raw data are presented in S7 Fig. (B) Flow cytometry-based quantification of infected cells (GFP^+^) among live (propidium iodide^-^) BMDMs after 10h of infection with GFP-expressing *F*. *novicida* at a MOI of 10. (C) Quantification of bacterial loads in single cells after 10h of infection with GFP-expressing *F*. *novicida* at a MOI of 10, assessed by high-resolution microscopy in flow and presented as a comparison of both genotypes, with bacteria-per-cell values grouped by increments of 10. (D) Immunofluorescence of BMDMs infected for 10h with GFP-expressing *F*. *novicida* at a MOI of 1 (stained with DAPI (blue) and with an antibody to *F*. *novicida* (red); scale bars: 5 μm. (E) GFP intensity was quantified in single GFP-expressing *F*. *novicida* in BMDMs from the indicated genotypes primed or not overnight with 100 U/ml of IFN-γ and infected for 10 h. Cumulative data from two independent experiments are shown. The GFP intensity of single bacteria was normalized in each experiment to the average intensity of single bacteria in unprimed WT BMDMs. Dots represent the normalized GFP intensity of a single bacteria, the bar represents the mean. Fluorescence of ≈300 bacteria per sample and per experiment was analyzed. The dotted lines indicate the average intensity in unprimed WT BMDMs (100%) and the threshold set to quantify GFP^low^ bacteria (80%). Data are representative of two (C, D, F) or at least three (A, B) independent experiments. (A, B, F) Mean and s.d. of triplicate wells are shown. One-way ANOVA analysis with Tukey's correction for multiple comparisons (A-B, F) were performed. The distribution of GFP intensity in the different sample was analyzed using Komogorov-Smirnov test with Bonferroni correction.

The high IFN-γ levels observed in *Gbp*^chr3^-KO mice in the mouse model of tularemia coupled to the inability of *Gbp*^chr3^-KO mice to control *F*. *novicida* burden (Fig 1) led us to investigate if GBPs contribute to the IFN-γ-mediated growth restriction observed *in vitro* [45]. As previously described [13,15,24,35], we observed a robust *F*. *novicida* replication in unprimed macrophages (Fig 5A corresponding to the Raw data presented in S7 Fig), which was partially controlled in a GBP-dependent manner by the inflammasome. IFN-γ priming led to *F*. *novicida* killing as visualized by a net decrease in the recovered intracellular colony forming units (Fig 5A) as soon as 6 h PI. Bacterial killing was highly dependent on GBPs. Indeed, *F*. *novicida* was not killed in IFN-γ-primed *Gbp*^chr3^-KO macrophages, but robustly replicated by a 40-fold over 12 h of infection. We did not observe any substantial effect of IFN-γ on phagosomal rupture (S8 Fig) suggesting that IFN-γ does not act by restricting *F*. *novicida* access to its cytosolic niche. This finding is consistent with a previous study demonstrating a direct activity of IFN-γ on cytosolic *F*. *tularensis* [45]. Furthermore, the IFN-γ-mediated bacterial growth inhibition was independent of the NADPH oxidase and of the IFN-γ-inducible NO synthase (iNOS also known as NOS2), two immune effectors described to act downstream of GBPs [46] (S9C–S9E Fig). In WT macrophages, the GBPs-mediated bacterial killing was difficult to segregate from the antibacterial effects of host cell death due to the very rapid inflammasome activation (see Fig 2B). Yet, bacterial killing was also observed in IFN-γ-primed *Asc*^*-/-*^ macrophages infected for 6 h at a MOI of 1 in the absence of substantial pyroptotic and apoptotic cell death. Similarly, IFN-γ-mediated blockage in bacterial replication was observed in *Casp1/Casp11*^*-/-*^ and *Aim2*^*-/-*^ macrophages (S9A Fig) suggesting that intracellular bacterial killing is independent of canonical and non-canonical inflammasomes. To assess whether IFN-γ-mediated inhibition of bacterial growth was dependent or independent of cell death we took advantage of flow cytometry using GFP-expressing *F*. *novicida* and propidium iodide to exclude dead cells. In the absence of IFN-γ priming, replication was observed in ≈30% of live *Asc*^*-/-*^ and ≈50% *Gbp*^chr3^-KO macrophages (as determined by the number of propidium iodide^-^ GFP^+^ cells). In the presence of IFN-γ, this number dropped to less than 4% in *Asc*^*-/-*^ macrophages indicating a robust bacterial growth restriction in these cells. In contrast, a large number of *Gbp*^chr3^-KO macrophages (>40%) sustained *F*. *novicida* replication despite IFN-γ priming (Fig 5B). These results suggest that this anti-bacterial GBP-dependent mechanism proceeds independently of cell death at least in *Asc*^*-/-*^ BMDMs.

To quantify *F*. *novicida* replication in a large number of macrophages, we analyzed infected macrophages using high-resolution microscopy in Flow (ImagestreamX). This single cell quantification technique further demonstrated that in the absence of *Gbp*^chr3^, IFN-γ was almost ineffective to control bacterial replication. Indeed, in the presence of IFN-γ, there was a 100-fold increase in the number of *Gbp*^chr3^-KO macrophages permissive for bacterial replication (containing more than 100 bacteria) compared with that of *Asc*^*-/-*^ macrophages (Fig 5C, S9F Fig). Representative immunofluorescence images of this striking phenotype are presented in Fig 5D. While this difference was exacerbated in the presence of IFN-γ and as previously noticed using *Aim2*^*-/-*^ macrophages [13], we observed that in unprimed macrophages, GBPs also controlled in an ASC-independent manner the bacterial burden.

GBP5-associated *F*. *novicida* loose their GFP expression, which has been associated with a loss of bacterial viability [15]. We thus quantified GFP intensity in single bacteria in propidium iodide-negative BMDMs as a surrogate marker of bacterial viability/metabolic activity (Fig 5E). In WT macrophages, IFN-γ treatment led to a significant reduction in the average GFP intensity (-10.2%) of intracellular *F*. *novicida* with a large increase (30%, n = 640) in the number of bacteria expressing low GFP levels (as defined by a GFP intensity <80% of the average intensity of bacteria in unprimed WT macrophages). Similarly, IFN-γ priming significantly reduced the average GFP intensity of bacteria in *Asc*^*-/-*^ BMDMs (-8.4%) with a large increase (+19%, n = 843) in the number of low GFP-expressing bacteria. Surprisingly, IFN-γ priming had a paradoxical effect on bacterial GFP expression in *Gbp*^chr3^-KO BMDMs with an increase in the average GFP intensity (+20.6%) and a strong decrease in the number of low GFP-expressing bacteria (-30%, n = 658). While this paradoxical increase (also visible in Fig 5D) remains to be understood, this experiment demonstrates that IFN-γ-induced GBPs in live macrophages affect the metabolic activity of bacteria. The decrease GFP expression observed in the presence of IFN-γ and GBPs is likely due to GBP-mediated antibacterial activity although we were unable to directly quantify bacteriolysis in single cells. Importantly, the antibacterial activity of IFN-γ against *F*. *tularensis* live vaccine strain (LVS) was also fully dependent on *Gbp*^chr3^ extending our results to an attenuated strain of the *F*. *tularensis* species (Fig 5F). While an IFN-γ-dependent GBPs antibacterial activity was clearly observed upon LVS infection (Fig 5F; 23x fold reduction in WT BMDMs upon IFN-γ treatment), LVS sustained a substantial replication (≈100x) in IFN-γ-primed BMDMs. More strikingly, we were unable to observe a robust IFN-γ-mediated growth restriction of *F*. *tularensis* SCHU S4 in BMDMs (S10 Fig). These results suggest that *F*. *tularensis* strains have evolved mechanisms to avoid, at least partially, IFN-γ-mediated GBPs-dependent antibacterial activity.

The synergistic roles of GBP2 and 5 in promoting the antibacterial effect of IFN-γ could be demonstrated in J774.1 macrophage-like cells using CRISPR/cas9 (S11 Fig). This result rules out that the extensive replication observed in *Gbp*^chr3^-KO BMDMs despite the priming with IFN-γ could be due to a non-specific defect of *Gbp*^chr3^-KO BMDMs associated with their large genomic deletion. This is well in line with control experiments previously performed on *Gbp*^chr3^-KO mice demonstrating normal induction of IFN-inducible genes and normal susceptibility/resistance to certain pathogens including *L*. *monocytogenes* [8,16]. Altogether, these findings demonstrate that, *in vitro*, in infected macrophages, the anti-bacterial function of IFN-γ, a cytokine known to induce a large number of antibacterial effectors relies almost exclusively on *Gbp*^chr3^.

### rIFN-γ administration partly rescues the in vivo antimicrobial function of *Asc*^-/-^ mice but fails to complement *Gbp*^chr3^ deficiency

**Fig 6:**
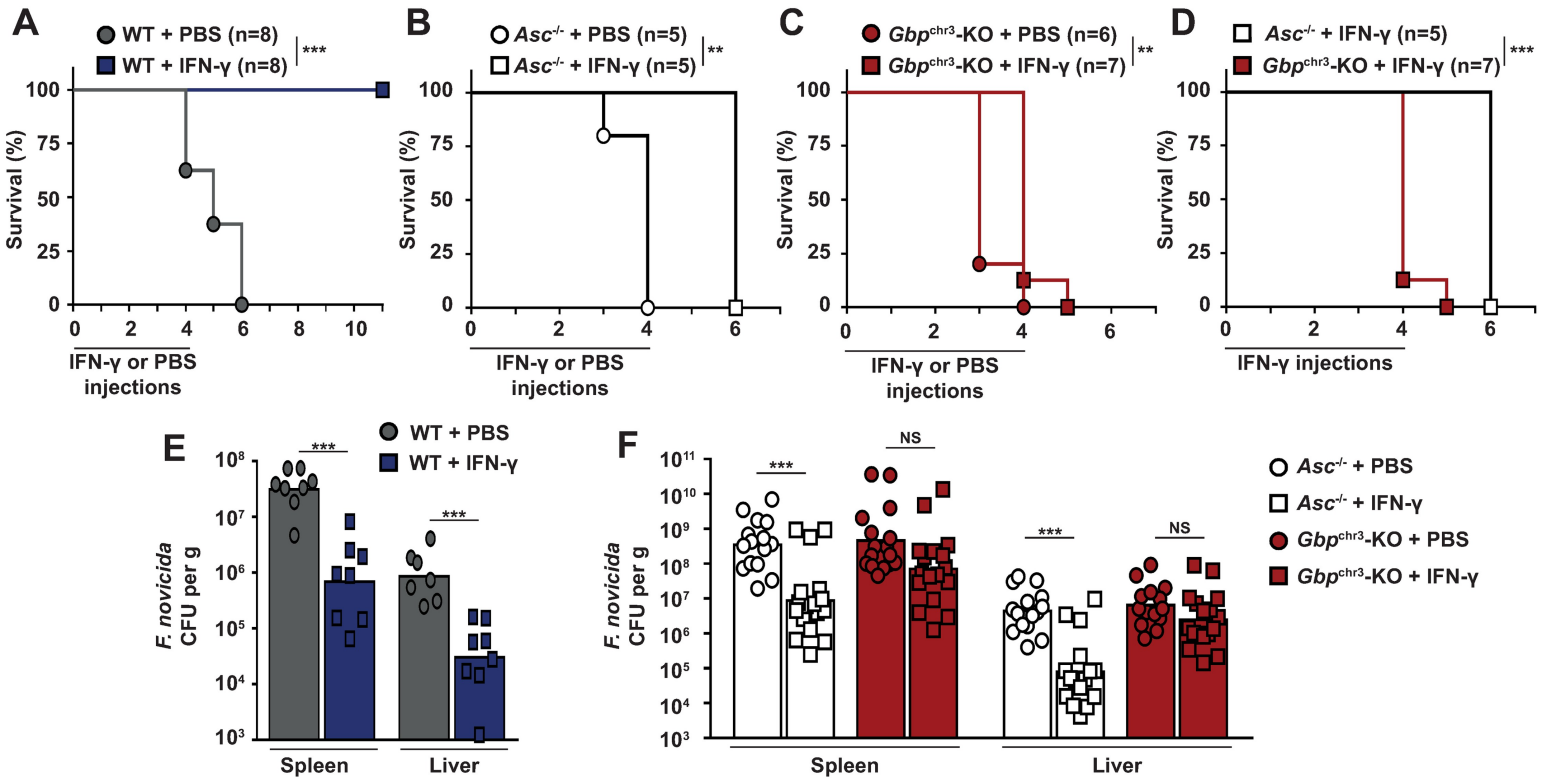
rIFN-γ administration largely rescues the *in vivo* antimicrobial function of *Asc*^*-/-*^ mice but fails to complement *Gbp*^chr3^ deficiency. (A-D) Survival of mice treated by daily intraperitoneal injection of PBS or 10^5^ U of rIFN-γ during 5 days after sc inoculation with 5×10^4^ *F*. *novicida* (A) or 5×10^3^ *F*. *novicida* (B-D). (A-C) effect of IFN-γ treatment in each genotype, (D) comparison of IFN-γ -treated *Asc*^*-/-*^ and *Gbp*^chr3^-KO mice. (E-F) Bacterial burden in the liver and spleen 2 days after sc inoculation with 5×10^4^ *F*. *novicida* (E) or or 5×10^3^ *F*. *novicida* (F). Each symbol represents the value of an individual mouse, geometric mean is shown. Data are representative of two independent experiments. Log-rank Cox-Mantel test (A-D), Mann-Whitney (E) and Kruskal-Wallis analysis with Dunn's correction (F) were performed.

GBPs have been tightly linked to inflammasome complexes [10–16]. Yet our *in vitro* data clearly demonstrate that GBPs have potent inflammasome-independent antimicrobial functions. Particularly, *in vitro*, GBPs are the main IFN-γ antimicrobial effectors. In contrast, in the presence of IFN-γ, the inflammasome complex seems largely facultative to control *F*. *novicida* replication. ASC is required *in vivo* to induce IFN-γ via IL-18 release [27,28,47]. To further explore these potential differences *in vivo*, we administered rIFN-γ to *F*. *novicida*-infected mice. Early rIFN-γ administration allowed WT mice to survive *F*. *novicida* infection (Fig 6A). rIFN-γ administration clearly extended *ASC*^*-/-*^ mice survival (Fig 6B) and strongly delayed *Asc*^*-/-*^ mice weight loss (S12 Fig), while it had a very moderate (although statistically significant) effect on *Gbp*^chr3^-KO mice survival and weight loss (Fig 6C and 6D, S12 Fig). To assess the functional links *in vivo* between IFN-γ, GBPs or the inflammasomes and their impact on bacterial replication, we analyzed the bacterial burden in the spleen and the liver at 48h PI following rIFN-γ injection at day 0 and day 1 PI. rIFN-γ was highly efficient to control the bacterial burden in both WT (Fig 6E) and *Asc*^*-/-*^ mice (Fig 6F). In contrast, upon IFN-γ injection in *Gbp*^chr3^-deficient mice, there was no statistical reduction in the bacterial burden in the liver and the spleen (4-fold reduction in the average splenic burden in *Gbp*^chr3^-KO mice versus a 40-fold reduction in *Asc*^*-/-*^ mice) (Fig 6F). These results demonstrate that, as observed in infected macrophages, the antibacterial action of IFN-γ is mostly mediated by GBPs *in vivo* and is largely independent of the canonical inflammasomes.

## Discussion

With the recent discoveries of the tight links between the inflammasome complexes and the GBPs, it became unclear whether most antimicrobial functions of GBPs in infected macrophages were mediated by various inflammasome complexes or were independent of the inflammasomes [48]. Indeed, *Gbp*^chr3^- and inflammasome-deficient mice are similarly highly susceptible to *F*. *novicida* infection. One of the current models to explain the link between GBPs and the inflammasomes positions GBPs as the molecular platform promoting the inflammasome supramolecular complex [2,3,48,49]. This model is strongly supported by evolution since GBPs from jawed fish display inflammasome-related CARD domains [14]. During *F*. *novicida* infection, we and other have previously demonstrated that GBPs are required to trigger AIM2 inflammasome activation while they are dispensable upon direct delivery of DNA into the host cytosol [13,15]. GBPs, in cooperation with IRGB10, were demonstrated to participate in *F*. *novicida* lysis into the host cytosol [13,15,16] suggesting that GBPs mostly act to release bacterial DNA into the host cytosol. The polymeric nature of DNA might alleviate the requirement for host factors to promote AIM2 inflammasome activation. Indeed, cytosolic dsDNA could provide the scaffold for AIM2 oligomerization and subsequent inflammasome activation [50,51].

In this work, we demonstrate that GBPs are required not only for the AIM2 canonical inflammasome activation but for most of the programmed cell death pathways that can take place in *F*. *novicida*-infected macrophages. *In vitro*, in *F*. *novicida*-infected WT murine BMDMs, so far the only described cell death pathway was the AIM2 inflammasome. Yet, using various knock-out macrophages, we have demonstrated here that several canonical and non-canonical inflammasome complexes can be active during *F*. *novicida* infection. These alternative pathways were revealed thanks to the use of *Aim2*^*-/-*^ BMDMs and are masked in WT macrophages. Interestingly, Harton and colleagues recently demonstrated that *F*. *tularensis* strains, in contrast to *F*. *novicida*, elicit NLRP3 inflammasome activation in BMDMs [52]. This result suggests that *F*. *tularensis* has evolved to escape AIM2 inflammasome activation while the delayed NLRP3-dependent sensing is conserved in response to various *Francisella* species. The identification of a caspase-11-dependent role in mediating *Aim2*^-/-^ BMDMs death and IL-1β release in the absence of AIM2 was unexpected. Indeed, the direct delivery of *Francisella* LPS into BMDMs cytosol does not activate caspase-11 due to its underacylated structure ([36]). It is still unclear whether the delayed caspase-11-dependent activation observed in *Aim2*^-/-^ BMDMs is due to sensing of *F*. *novicida* LPS or of another endogenous or bacterial ligand. The large number of inflammasome complexes activated upon *F*. *novicida* infection is reminiscent of what have been observed during infection with other intracellular bacteria [11,53,54], although *F*. *novicida* infection of murine macrophages is somewhat unique due to its high dependence on AIM2 [18]. Remarkably, IFN-γ-induced GBPs are required to trigger all these pathways suggesting that they either act upstream of the inflammasomes or that they have conserved mechanisms to facilitate activation of several (AIM2, NLRP3, caspase-11) inflammasome sensors. Such facilitation of the activation of multiple inflammasomes by GBPs has been previously observed upon *Chlamydia* infection [11]. Yet, GBPs-mediated control of *Chlamydia*-mediated cell death was only partial in contrast to what we observed upon *F*. *novicida* infection. Importantly, *Asc*^*-/-*^ (and *Casp1/Casp11*^*-/-*^) BMDMs died by apoptosis as demonstrated by morphological and molecular analyses (Fig 4, S1 and S2 Movies)[35] indicating that GBPs control both pyroptotic and apoptotic pathways. Indeed, processing of caspase-8, 9 and 3 and DEVDase activity were clearly visible in *Asc*^*-/-*^ BMDMs but absent in *Asc*^*-/-*^*Gbp*^chr3^-KO BMDMs (S6D and S6E Fig). The GBP-dependent pathway leading to apoptotic caspase activation in *Asc*^*-/-*^ BMDMs is still unclear. Antibiotic-mediated bacteriolysis of another cytosolic pathogen (*Shigella flexneri*) triggers massive caspase-9-dependent apoptosis of epithelial cells [55]. Based on the antibacterial role of GBPs ([13,15,16], this work), we speculate that GBPs-mediated action releases a PAMPs that directly or indirectly triggers apoptosis in the absence of the inflammasome adaptor ASC. The concurrent maturation of both caspase-8 and caspase-9 suggests activation of both extrinsic and intrinsic (e.g. mitochondrial) apoptotic pathways. This dual activation may be due to the cross-activation of the mitochondrial intrinsic apoptosis pathway following cleavage of Bid by caspase-8 [56]. Yet, we cannot exclude a direct GBPs-dependent induction of mitochondrial dysfunction such as the one occurring in *S*. *flexneri*-infected cells of non-myeloid lineages [57]. IRGs can lyse *T*. *gondii* vacuole leading to parasite permeabilisation and a caspase-1-independent necrotic death in mouse embryonic fibroblats [58]. Due to the diversity of cell death pathways controlled by GBPs and IRGs, we favor the hypothesis that these IFN-induced GTPases act as PAMPs-shedders to release/uncover various microbial cell death-activating ligands. Future studies are needed to establish the pathways linking GBPs to the different cell death pathways.

IFN-γ is the most potent cytokine against intracellular bacteria due to its ability to induce hundreds of genes promoting host defense [1]. Remarkably, our data indicate that the antimicrobial action of IFN-γ against *F*. *novicida* and *F*. *tularensis* Live Vaccine Strain is almost exclusively dependent on GBPs. While it was previously known that IFN-γ could restrict cytosolic *Francisella* growth independently of cell death, reactive oxygen or nitrogen species, autophagy and IDO-mediated tryptophan degradation [45], the mechanisms responsible for this growth restriction were unknown. The IFN-γ-mediated *F*. *novicida* growth restriction is independent of caspase-1 and caspase-11 (S3 Fig). It thus differs from the recently described mechanisms responsible for growth inhibition of cytosolic *Salmonella* [59]. Interestingly, the highly virulent *F*. *tularensis* SCHU S4 largely escaped IFN-γ-mediated antibacterial activity in BMDMs (S10 Fig). It remains unclear whether this escape is the result of an active process or due to the failure of innate immune sensors to detect/recognize cytosolic *F*. *tularensis* SCHU S4.

In a concurrent work, Kanneganti and colleagues identified IRGB10 as an IFN-inducible GTPase recruited onto cytosolic *F*. *novicida* and required to lyse the bacterium and trigger AIM2 inflammasome activation [16]. IRGB10 recruitment is abolished in *Gbp*^chr3^-KO macrophages indicating that GBPs and IRGB10 may act together and that the latter protein is likely involved in IFN-γ-mediated growth restriction in murine macrophages. In contrast to GBPs [6], IRGs (with the exception of the constitutively expressed IRGM) are absent in humans [60]. Yet, IFN-γ priming efficiently restricts cytosolic *Francisella* growth in human macrophages ([45], S13 Fig) indicating that IRGs are facultative for the IFN-γ-mediated antimicrobial role. While our results identify that GBPs are required for IFN-γ-mediated killing of cytosolic bacteria, other host factors are likely involved upstream of GBPs to facilitate GBPs targeting onto cytosolic bacteria. The molecular mechanisms sustaining IFN-γ-dependent GBPs-mediated antibacterial activity remains to be understood.

## Materials and methods

### Ethics statement

All animal experiments were reviewed and approved by the animal ethics committee (CECCAPP, Lyon) of the University of Lyon, France under the protocol number #ENS_2012_061, #ENS_2014_017 and #ENS_2017_002 and in strict accordance with the European regulations (#2010/63/UE from 2010/09/22) and the French laws ("Décret n 2013–118 du 1er février 2013 relatif à la protection des animaux utilisés à des fins scientifiques" and "Arrêté ministériel du 1er février 2013 relatif à l'évaluation éthique et à l'autorisation des projets impliquant l'utilisation d'animaux dans des procédures expérimentales").

### Mice

*Gbp*^chr3^-KO, *Nos2*^*–/–*^, *Cybb*^*–/–*^, *Casp1*^*–/–*^*/Casp11*^*–/–*^(a.k.a caspase-1 knockout), *Asc*^*–/–*^, *Aim2*^*–/–*^, *Nlrp3*^*–/–*^mice, all in the C57BL/6, have been previously described [8,24]. Double knock-out mice (*Nlrp3*^*-/-*^*Aim2*^*-/-*^ and *Asc*^*-/-*^ *Gbp*^chr3^-KO) were generated in the framework of this project. The presence of the functional C57BL/6 caspase-11 was verified by PCR amplification of exon 7 boundaries followed by sequencing [37]. Mice were bred at the PBES (Lyon, France).

### Mouse infections

Age- and sex-matched animals (6–10 weeks old) were infected subcutaneously with 5x10^3^ or 5x10^4^ or 4x10^5^ CFU of *F*. *novicida* in 100 μl PBS (as indicated in the figure legends). When applicable, 10^6^ U/ml of rIFN-γ was injected intraperitonealy in 100 μl PBS. Blood was collected by retro-orbital bleeding at 16 h post-infection or intra-cardiac puncture at 48 h post-infection. Animals were sacrificed at the indicated time point post-infection. Mice were examined twice daily for signs of severe infection and euthanized as soon as they displayed signs of irreversible morbidity or as soon as weight loss exceeded 20%.

### Bacterial strains and plasmids

*F*. *novicida* strain U112, its isogenic ΔFPI mutant [61] and *F*. *tularensis* subspecies *holarctica* Live Vaccine Strain (LVS) were used. When applicable, strains were transformed with pKK219-GFP [62].

### Cell culture and infections

Preparation and culture of BMDMs were performed as previously described [63]. BMDMs were differentiated in DMEM medium (Invitrogen) with 10% v/v FCS (Thermo Fisher Scientific), 10% MCSF (L929 cell supernatant), 10 mM HEPES (Invitrogen), 5% Sodium pyruvate. 1 day before infection, macrophages were seeded into 6-, 24-, or 96-well plates at a density of 1.25x10^6^, 2.5x10^5^, or 5x10^4^ per well. When applicable macrophages were pre-stimulated with 100ng/ml Pam_3_CSK_4_, LPS O111:B4 (InvivoGen) or 100u/ml mIFN-β or mIFN-γ (immunotools). For infections with *F*. *novicida*, bacteria were grown overnight in TSB supplemented with 0.1% (w/v) cysteine at 37°C with aeration. The bacteria were added to the macrophages at the indicated MOI. The plates were centrifuged for 15 min at 1500 g and placed at 37°C for 60 min. Cells were washed and fresh medium containing 10 μg.ml^-1^ gentamycin (Invitrogen) was added. For LVS, cells were infected for 2 h at an MOI of 0.4, washed and incubated in the presence of gentamicin at 5 μg.ml^-1^.

### Replication assay

For *F*. *novicida* intracellular replication assay, macrophages were lysed with 1% (w/v) saponin (Sigma) in water for 5 min. Dilution, plating on TSA supplemented with 0.1% (w/v) cysteine and counting was performed using the easySpirale Dilute (Interscience). For LVS replication assay, cells were lysed in PBS with 0.1% deoxycholate, serially diluted in PBS and plated on modified GC-agar base plates.

### siRNA knockdown

Gene expression knockdown was done using GenMute (SignaGen laboratories) and siRNA pools (siGenome, Dharmacon). Briefly, wild-type BMDMs were seeded into 24-, or 96-well plates at a density of 1.5x10^5^ or 3x10^4^ per well. siRNA complexes were prepared at 25 nM in GenMute Buffer according to the manufacturer’s instructions for forward knockdowns. siRNA complexes were mixed with BMDMs medium and added onto the cells. BMDMs were infected with *F*. *novicida* at an MOI of 10:1 after 48 h of knockdown and analyzed for inflammasome activation as outlined below. siRNA pools included: Aim2 (M-044968-01), Caspase-11 (that is, Casp4) (M-042432-01), Mefv (M-048693-00), Nlrp3 (M-053455-01), Caspase-1 (M-048913-01) and NT (non-targeting) pool 2 (D-001206-14).

### Cytokine measurement and cell death assays

IL-1β and IL-18 were measured by ELISA (R&D systems and platinum ebioscience, respectively). Cell viability was determined by the CellTiter-Glo Luminescent Cell Viability Assay (Promega). Global cell death kinetics was monitored in BMDMs by assessing in real time incorporation of propidium iodide (used at 5 μg/ml) through measurement of fluorescence emission at 635 nm every 15 min on a microplate reader (Tecan, see. S2A Fig for the sensitivity of the technique). When indicated triton X100 (Sigma) at 1% (v/v) was added at the end of the kinetics to further control cell death/viability. Area under the curve were computed using Prism software (GraphPad) to obtain a single quantitative readout of the full kinetics as recently described [64]. Gliotoxin (Enzo Pharma) and Nigericin (Sigma) were used at 5 μM, the latter after a 3 h priming with LPS at 100 ng/ml. Single cell death kinetics was determined using an automated time-lapse video microscope (Arrayscan high-content system, Thermo Fisher Scientific). Image analyses of four sparse fields per well were performed using the HCS studio analysis software. Wheat-germ agglutinin (WGA)-labelled BMDMs were used. Individual dead cells were detected and numerated based on the propidium iodide fluorescence staining and normalized to the total number of cells numerated through the vital Hoechst staining at time 0. WGA intensity in propidium iodide positive cells was calculated to quantify cell retraction using HCS studio software. CO2-independent medium (ThermoFisher Scientific) was used for all cytokines dosage and cell death kinetics.

### Protein lysates and caspase activity assay

Following BMDMs infection, protein extracts were obtained by lysing cells in the following buffer (10 mM Hepes/KOH, 2mM EDTA, 0.1% CHAPS, 250 mM sucrose, 5mM dithiothreitol). Samples were clarified by centrifugation at 4°C, 13 000g for 15 minutes. Protein concentration was determined using Bradford method (Bio-Rad). Fluorimetric analysis of caspase-3/7 activity was performed as previously described [35] by incubating protein extracts (4 μg/sample) with Ac-DEVD-AFC (Enzo pharma, ALX-260-037) at 40 μM final concentration. Fluorescent reading over 3 h was performed on a fluorimeter (Tecan).

### Immunoblotting

Blotting was done as described before using 15 to 20 μg of protein sample per lane depending on the antibodies [13]. Antibodies used were rabbit anti-GBP2 and rabbit anti-GBP5 (1:1,000; 11854-1-AP/13220-1-AP; Proteintech), anti-caspase-8 (1:2,000; Enzo pharma; ALX-804-447), anti-caspase-9 (1:2,000; MBL; M054), anti-caspase-3 and anti-cleaved caspase-3 (1:1,000; Cell signaling Technologies; #9662 and #9661, respectively). Cell lysates were probed with anti-β-actin antibody (Sigma) at 1:2,000.

### Immunofluorescence and GFP quantification in single bacteria

Macrophages were seeded on glass coverslips and infected as described above. At the desired time, cells were washed 3 times with PBS and fixed with 4% paraformaldehyde for 15 min at 37°C. Following fixation, coverslips were washed and the fixative was quenched with 0.1 M glycine for 10 min at room temperature. Coverslips were stained with primary antibodies at 4°C for 16 h, washed with PBS, incubated for 1 h with appropriate secondary antibodies at room temperature (1:500, AlexaFluor, Invitrogen), washed with PBS and mounted on glass slides with Vectashield containing 6-diamidino-2-phenylindole (DAPI) (Vector Labs). Antibodies used were chicken anti-*Francisella* (1:1000, a gift from D. Monack). Coverslips were imaged on a Zeiss LSM710.

Quantification of GFP in GFP-expressing *F*. *novicida* was performed using an automated process in ImageJ (NIH, USA). The threshold was adjusted using the moments-preserving thresholding method with the dark background option. Binary watershed process was used to separate individual bacteria. Fluorescence intensity quantification was restricted to individual particle of 0.2 to 2 μm^2^ with a circularity comprised between 0.5 and 1.

### Flow cytometry and microscopy in flow

For assessment of bacterial replication by flow cytometry, macrophages seeded onto non-tissue culture-treated plates were infected as described above with GFP-expressing *F*. *novicida* strains. At desired time, cells were lifted with trypsin and immediately analyzed by Flow cytometry on a Canto 2 cytometer (BD biosciences). Dead cells were excluded based on staining with propidium iodide.

For the microscopy in flow experiments, macrophages infected with GFP-expressing bacteria were fixed in PFA 4% and analyzed on ImageStream X mark II (Amnis, EMD-Millipore) using the Inspire software with the Extended depth of field (EDF) function activated to increase the spot counts accuracy. Images of single cells were analyzed with the Ideas Software (Amnis, EMD-Millipore) as previously described [13] to quantify the number of bacteria per cell.

### Statistical analysis

Statistical data analysis was done using Prism 5.0a (GraphPad Software, Inc.). To evaluate the differences between three selected groups or more (cell death, cytokine release, FACS, CFU and immunofluorescence-based counts) one-way ANOVA analysis was performed with Tukey's correction for multiple analysis. Komogorov-Smirnov test was used to compare the cell distribution as determined by Imagestream and the distribution of GFP intensity in single bacteria. P values were adjusted for multiple comparisons with the Bonferroni correction approach. Animal experiments were evaluated using Kruskal-Wallis analysis with Dunn's correction except when only two groups were present in the analysis, Mann-Whitney analysis was performed. Survival experiment was analyzed thanks to log-rank Cox-Mantel test. In figures NS indicates ‘not significant’, P values are given according to the following nomenclature: *P<0.05; **P<0.01; ***P<0.001.

## Supporting information

S1 Material and Methods Supplemental methods including CCF4 measurements, SCHU S4 infection, real-time PCR, CRISPR/Cas9-mediated knock-out procedures. (DOCX)

S1 Fig IFN-γ priming induces a quantitative shift in GBPs levels. (A) GBP2, (B) GBP5 (C) ProIL-1β mRNA levels, (D) GBP2, GBP5 and β-actin protein levels were analyzed by qRT-PCR (A-C) or western blotting analysis (D) in BMDMs from the indicated genotypes infected with *F*. *novicida* at a MOI of 10 for 4 h or treated for 16 h with 100 U/ml of IFN-β, IFN-γ or 100 ng/ml of Pam_3_CSK_4_ (NT: not treated). (A-B) One-way ANOVA analysis was performed with Tukey's correction to compare GBP induction following IFN-β and IFN-γ priming. (A-D) One experiment representative of two independent experiments, (A-C) mean and standard deviations are shown.
(TIF)

S2 Fig Controls related to the real time measurement of propidium iodide incorporation/fluorescence. (A) WT BMDMs were seeded at different cell density as indicated and treated with Triton X100 (1% v/v final) in the presence of propidium iodide. Cell death was analyzed in real time by quantifying propidium iodide fluorescence every 5 minutes. (B) IFN-γ-primed BMDMs from the indicated genotypes were infected with *F*. *novicida* at a MOI of 10. Cell death was analyzed by quantifying propidium iodide fluorescence every 15 minutes. At 26 h post-infection, TX-100 (1% v/v final) was added leading to a strong increase in fluorescence in *Gbp*^chr3^-KO BMDMs but not in WT nor *Asc*^-/-^ BMDMs confirming that most *Gbp*^chr3^-KO BMDMs had an intact (propidium iodide-impermeant) plasma membrane before TX-100 addition in contrast to most WT and *Asc*^*-/-*^ BMDMs. One experiment representative of two (A) to at least three (B) independent experiments is shown.
(TIF)

S3 Fig Time-lapse microscopy demonstrates that *F*. *novicida*-infected BMDMs trigger, in a hierarchical manner, various GBPs-dependent cell death pathways. (A,B) Cell death was monitored at single cell level and in real time using automated microscopy and propidium iodide. BMDMs of the indicated genotypes were primed (B) or not (A) with rIFN-γ (100 U/ml) for 16h before infection with *F*. *novicida* at an MOI of 10. Automated image analysis was used to quantify the percentage of dead cells at each time points of the kinetics. (C) The area under the curve (corresponding to the above kinetics from 1.5 to 20 h post-infection) was computed. One-way ANOVA analysis was performed with Tukey's correction. (A-C) One experiment representative of two independent experiments is shown.
(TIF)

S4 Fig Knock-down of the expression of inflammasome components demonstrated hierarchical activation of several cell death pathways. (A) Knock-down efficiency and specificity was determined by qRT-PCR at 48 h post-transfection in BMDMs. The specific transcript levels were normalized to β-actin transcript level and rationalized with the corresponding transcript level in BMDMs treated with a non-targeting (NT) siRNA. (B) *Aim2*^*-/-*^ BMDMs transfected with the indicated siRNA were infected with *F*. *novicida* at a MOI of 10 and cell death was monitored by measuring propidium iodide fluorescence at 17 h PI. (C) WT BMDMs transfected with the indicated siRNA were infected with *F*. *novicida* at a MOI of 10 and cell death was monitored in real time by measuring propidium iodide fluorescence. (D) The area under the curve corresponding to the (C) kinetics from 1 to 20h is shown. NI: Non-infected. (B-D) One-way ANOVA analysis was performed with Tukey's correction. (A-D) One experiment representative of three independent experiments is shown.
(TIF)

S5 Fig IFN-γ modulates the kinetics of IL-1ß release in *Gbp*^chr3^-KO BMDMs. BMDMs from the indicated genotypes were infected or not with *F*. *novicida* at a MOI of 10 after priming or not with IFN-γ (100u / ml 16 h). At 10 h post-infection (A) or 24 h post-infection (B) IL-1ß concentrations were determined by ELISA. One experiment representative of three independent experiments with mean and standard deviations is shown. One-way ANOVA analysis was performed with Tukey's correction.
(TIF)

S6 Fig Comparison of the responses of macrophages doubly deficient in *Asc* and *Gbp*^chr3^ with that of macrophages with single deficiencies demonstrates that GBP and ASC control different pathways. BMDMs from the indicated genotypes (DKO corresponds to *Asc*^*-/-*^ *Gbp*^Chr3^-KO doubly-deficient macrophages) were infected or not with *F*. *novicida* at a MOI of 10 after priming (B) or not (A) with IFN-γ (100 U/ml, 16 h). (A, B) Real time propidium incorporation/ fluorescence, (C) area under the curve corresponding to the kinetics in A and B, (D) apoptotic caspases processing analysis by Western blotting, (E) DEVDase activity as determined using a fluorogenic caspase-3 substrate and (F) bacterial replication assay by CFU are shown. (C) The dotted vertical lines in Casp9 Western blot illustrate that the samples from a single original Western blot gel/ image were reorganized to fit the indicated order without any other image manipulation. The plain vertical line in Casp-3 Western blot illustrates that the samples from two Western blot gels run and analyzed side by side with the same exposure time are presented. The dotted horizontal lines in Casp8 and Casp3 Western blot indicate images from two different exposure times or from the use of two different primary antibodies (pro- and cleaved Casp3), respectively. The Western blots presented correspond to the ones presented in Fig 4D of the main manuscript. (A, B, C) one experiment representative of two independent experiments is shown. (C) One way ANOVA analysis with Tukey's correction for multiple tests was performed. (D-F) One experiment is shown. Mean and standard deviations are shown.
(TIF)

S7 Fig IFN-γ-induced GBPs control intracellular bacterial replication. (A-B) BMDMs from the indicated genotypes were primed or not overnight with 100 U/ml of IFN-γ. BMDMs were infected with (A) *F*. *novicida* or (B) *F*. *tularensis* LVS at a multiplicity of infection (MOI) of 1 and 0.4, respectively. Intracellular bacterial burden was assessed by determination of viable counts at the indicated times post-infection. The corresponding data expressed as Fold increase are presented in Fig 5.
(TIF)

S8 Fig IFN-γ does not modify substantially phagosomal rupture. BMDMs from the indicated genotypes were primed or not with 100 U/ml of IFN-γ for 16 h. BMDMs were infected with the indicated *F*. *novicida* strains at a multiplicity of infection (MOI) of 10. At 2 h post-infection, cells were incubated with the FRET substrate CCF4. Cytosolic ß-lactamase-mediated CCF4 hydrolysis (a marker of phagosomal permeabilization) was analysed by flow cytometry after gating on live (propidium iodide negative) cells. One experiment representative of two independent experiments is shown. One-way ANOVA analysis was performed with Tukey's correction.
(TIFF)

S9 Fig IFN-γ-induced GBPs control bacterial killing independently of inflammasomes, the NADPH oxidase and iNOS. (A-E) BMDMs from the indicated genotypes were primed or not overnight with IFN-γ (100 U/ml) and infected at a MOI of 1 with *F*. *novicida* (A-D) or at a MOI of 10 with GFP-expressing *F*. *novicida* (E). (A, D) Intracellular bacterial burden was assessed by determination of viable counts at 12 h. (A, C) Results were normalized with the viable counts detected at 2 h post-infection. The corresponding Raw data are presented in (B, D). (E) Flow cytometry-based quantification of infected (GFP^+^) cells among live BMDMs at 10 h post-infection. (F) Sample ImageStreamX images of BMDMs from the indicated genotypes, treated or not for 16 h with IFN-γ (100 U/ml) and infected at a MOI of 10 with GFP-expressing *F*. *novicida*.
(TIF)

S10 Fig Highly virulent *F*. *tularensis* SCHU S4 strain largely escapes IFN-γ-mediated growth restriction in BMDMs. WT BMDMs primed or not overnight with IFN-γ (100 U/ml) were infected at a MOI of 0.4 with *F*. *tularensis* SCHU S4 or *F*. *novicida* U112. Intracellular bacterial burden was assessed by determination of viable counts at 48 h. (A) Results were normalized with the viable counts detected at 2 h post-infection. (B) The corresponding Raw data are presented.
(TIF)

S11 Fig IFN-γ-induced GBP2 and 5 control bacterial killing in the J774.1 macrophage cell line as determined using CRISPR/Cas9 technology. Cas9-expressing J774.1 cells were transduced with non-targeting (NT) gRNA or gRNAs targeting the indicated gene(s). Following puromycin selection, the obtained cell lines primed or not with IFN-γ (100 U/ml for 16 h) were infected with GFP-expressing *F*. *novicida*. Live (propidium iodide-negative) cells were analyzed by flow cytometry at 14 h post-infection. One experiment representative of three independent experiments, mean and standard deviations are shown. One-way ANOVA analysis was performed with Tukey's correction.
(TIFF)

S12 Fig rIFN-γ administration protects WT and *ASC*^*-/-*^ and to a much lower extent *Gbp*^chr3^-*KO* mice from *F*. *novicida*-mediated weight loss. Weight loss of mice of the indicated genotypes (see associated Fig 6) treated by daily i.p injection of PBS or 10^5^ U of rIFN-γ during 5 days after s.c. inoculation with 5×10^4^ (A) or 5x10^3^ (B) *F*. *novicida*.
(TIF)

S13 Fig IFN-γ priming efficiently restricts cytosolic *F*. *novicida* growth in primary human macrophages. Flow cytometry-based quantification of live infected (PI^-^, GFP^+^) primary human macrophages from one healthy donor primed or not with hrIFN-γ (100 U/ml) of and infected for 16 h with GFP-expressing *F*. *novicida* strain U112 or the isogenic ΔFPI mutant at a MOI of 1. Mean and s.d. of triplicate wells are shown. Data are representative of two independent experiments.
(TIFF)

S1 Movie Cell death kinetics of unprimed BMDMs as assessed by time-lapse videomicroscopy. WGA-labeled WT, *Gbp*^chr3^-KO, *Casp1*^*-/-*^*Casp11*^*-/-*^ and *Asc*^*-/-*^ BMDMs were infected with *F*. *novicida* at a MOI of 1 in the presence of propidium iodide. Images were recorded every 30 minutes from 3 h PI to 23 h PI. Bright-field (top panels) and WGA (bottom panels) are shown. Note the increase in WGA intensity associated with cell retraction in *Casp1*^*-/-*^*Casp11*^*-/-*^ BMDMs previously demonstrated to die by apoptosis.
(AVI)

S2 Movie Cell death kinetics of IFN-γ-primed BMDMs as assessed by time-lapse videomicroscopy. IFN-γ-primed (100 U/ml for 16h) WGA-labeled WT, *Gbp*^chr3^-KO, *Casp1*^*-/-*^*Casp11*^*-/-*^ and *Asc*^*-/-*^ BMDMs were infected with *F*. *novicida* at a MOI of 1 in the presence of propidium iodide. Images were recorded every 30 minutes from 3 h PI to 23 h PI. Bright-field (top panels) and WGA (bottom panels) are shown.
(AVI)
