# Supplementary material for: IFN-γ extends the immune functions of Guanylate Binding Proteins to inflammasome-independent antibacterial activities during Francisella novicida infection
Source: PLoS Pathog. 2017 Oct 2;13(10):e1006630. doi: 10.1371/journal.ppat.1006630 (PMC5624647; doi:10.1371/journal.ppat.1006630)
Supplement: S1 Material and Methods — (DOCX) [file ppat.1006630.s001.docx]

**S1 Materials and Methods**

**CCF4 measurements.** Quantification of escape from vacuoles with the β-lactamase–CCF4 assay was performed following manufacturer's instructions (Life Technologies) as previously described [1]. The β-lactamase (Δ*bla*) mutant deleted in the FTN_1072 gene was previously described [2]. Macrophages seeded onto non-treated plates were infected for 1 h then were washed and then incubated for 1 h at room temperature in CCF4 in the presence of 2.5 mM probenicid (Sigma). The quantification of cells containing cytosolic *F. novicida* was performed on propidium iodide-negative (live) cells with excitation at 405 nm and detection at 450 nm (cleaved CCF4) or 510 nm (intact CCF4) by Flow cytometry.

**SCHU S4 infection**. Infection with *F. tularensis* SCHU S4 strain was performed in BSL3 laboratory as previously described [3] with the following modifications: BMDMs were used instead of Peritoneal exudate cells and a MOI of 0.4 was used.

**Real-time PCR.** The following primers were used for mRNA quantification: mAIM2 Forward (F): 5’AGTACCGGGAAATGCTGTTG3’, mAIM2 Reverse (R): 5’GCACCTGCACTTTGAATCAG3’, mNLRP3 F: 5’CCCTTGGAGACACAGGACTC3’, mNLRP3 R: 5’GAGGCTGCAGTTGTCTAATTCC3’, mMEFV F: 5’GGAGATGAGGGGATATGTGG3’, mMEFV R: 5’TGGATTTCTGTTTGTTTCAGGA3’, mCaspase-11 F: 5’TCCAGACATTCTTCAGTGTGGA3’, mCaspase-11R: 5’TCTGGTTCCTCCATTTCCAG3’, mβ-Actin F: 5’GTGGATCAGCAAGCAGGAGT3’, mβ-Actin R: 5’AGGGTGTAAAACGCAGCTCA3’. Experiments were performed with an iCycler (Bio-Rad) using SYBR green (Applied Biosystems) and standard protocols.

**CRISPR/Cas9-mediated Knock-out.** J774.1 murine macrophage-like cells were transduced using lentiviral particles produced in 293T cells using the plasmid LentiCas9-Blast (Gift from Feng Zhang, Addgene plasmid # 52962). Blasticidin selection was applied and clones were isolated by limit dilution method. A high Cas9-expressing clone was selected and transduced with lentiviral particles packaged with the pKLV-U6gRNA(BbsI)-PGKpuro2ABFP plasmid (Gift from Kosuke Yusa, Addgene plasmid # 50946). Two pairs of gRNAs (as indicated in the table below) were used for each knock-out. gRNA-expressing J774.1 cells were selected for 10 days in puromycin. Control experiments targeting GFP routinely give >90% of the cells demonstrating efficient knock-out. Production of lentiviral particle was performed in 293T cells by co-transfection of the following plasmids: pMD2.G (Gift from Didier Trono, Addgene plasmid #12259), psPAX2 (Gift from Didier Trono, Addgene plasmid #12260), and LentiCas9-Blast or pKLV-U6gRNA(BbsI)-PGKpuro2ABFP.

|  |  | Sens | Antisens |
| --- | --- | --- | --- |
| GBP2 | gRNA 1 | AAAGTTCCAGACAGAATTAG | CTAATTCTGTCTGGAACTTT |
|  | gRNA 2 | TCTTCTGTCAAGACTCTGTG | CACAGAGTCTTGACAGAAGA |
| GBP5 | gRNA 1 | ACACAGTAGTAACCTTGGCC | GGCCAAGGTTACTACTGTGT |
|  | gRNA 2 | CTCAAACATTCAATCTACCG | CGGTAGATTGAATGTTTGAG |
| GBP1/2b | gRNA 1 | CAAACTAGAGTGGATACAGG | CCTGTATCCACTCTAGTTTG |
|  | gRNA 2 | TGCTATCCAAAATCCTGTGG | CCACAGGATTTTGGATAGCA |
| Asc | gRNA 1 | CAGCTGCAAACGACTAAAGA | TCTTTAGTCGTTTGCAGCTG |
|  | gRNA 2 | CGCTCTTGAAAACTTGTCAG | CTGACAAGTTTTCAAGAGCG |
| gNT (non targeting) | gRNA 1 | GTTCGCGGGGGCTTCTATCA | TGATAGAAGCCCCCGCGAAC |
|  | gRNA 2 | TAACACGCACTCACGTCCGG | CCGGACGTGAGTGCGTGTTA |

Supplemental references:

1. Juruj C, V L, Pierini R, Perret M, Py BF, jamilloux Y, et al. caspase-1 activity affects AIM2 speck formation/stability through a negative feedback loop. Front Cell Infect Microbiol. 2013; 1–11.

2. Rigard M, Broms JE, Mosnier A, Hologne M, Martin A, Lindgren L, et al. Francisella tularensis IglG Belongs to a Novel Family of PAAR-Like T6SS Proteins and Harbors a Unique N-terminal Extension Required for Virulence. PLoS Pathog. 2016;12: e1005821. doi:10.1371/journal.ppat.1005821

3. Lindgren H, Stenman L, Tarnvik A, Sjostedt A. The contribution of reactive nitrogen and oxygen species to the killing of Francisella tularensis LVS by murine macrophages. Microbes Infect Inst Pasteur. 2005;7: 467–75. doi:10.1016/j.micinf.2004.11.020
