# Supplementary figures and images for: IFN-γ extends the immune functions of Guanylate Binding Proteins to inflammasome-independent antibacterial activities during Francisella novicida infection
Source: PLoS Pathog. 2017 Oct 2;13(10):e1006630. doi: 10.1371/journal.ppat.1006630 (PMC5624647; doi:10.1371/journal.ppat.1006630)

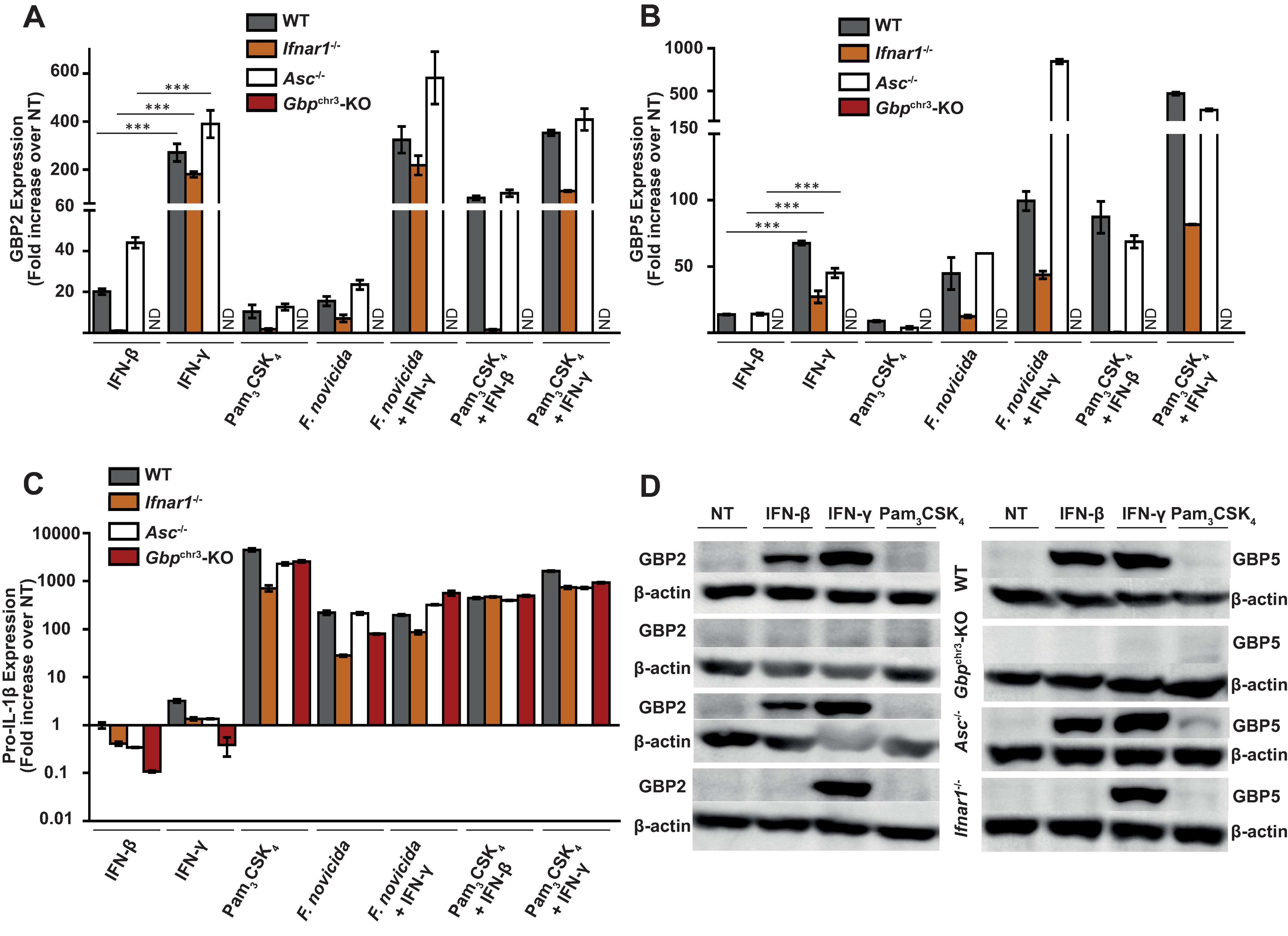

Supplement: S1 Fig — (A) GBP2, (B) GBP5 (C) ProIL-1β mRNA levels, (D) GBP2, GBP5 and β-actin protein levels were analyzed by qRT-PCR (A-C) or western blotting analysis (D) in BMDMs from the indicated genotypes infected with F. novicida at a MOI of 10 for 4 h or treated for 16 h with 100 U/ml of IFN-β, IFN-γ or 100 ng/ml of Pam3CSK4 (NT: not treated). (A-B) One-way ANOVA analysis was performed with Tukey's correction to compare GBP induction following IFN-β and IFN-γ priming. (A-D) One experiment representative of two independent experiments, (A-C) mean and standard deviations are shown. (TIF) [file ppat.1006630.s002.tif]

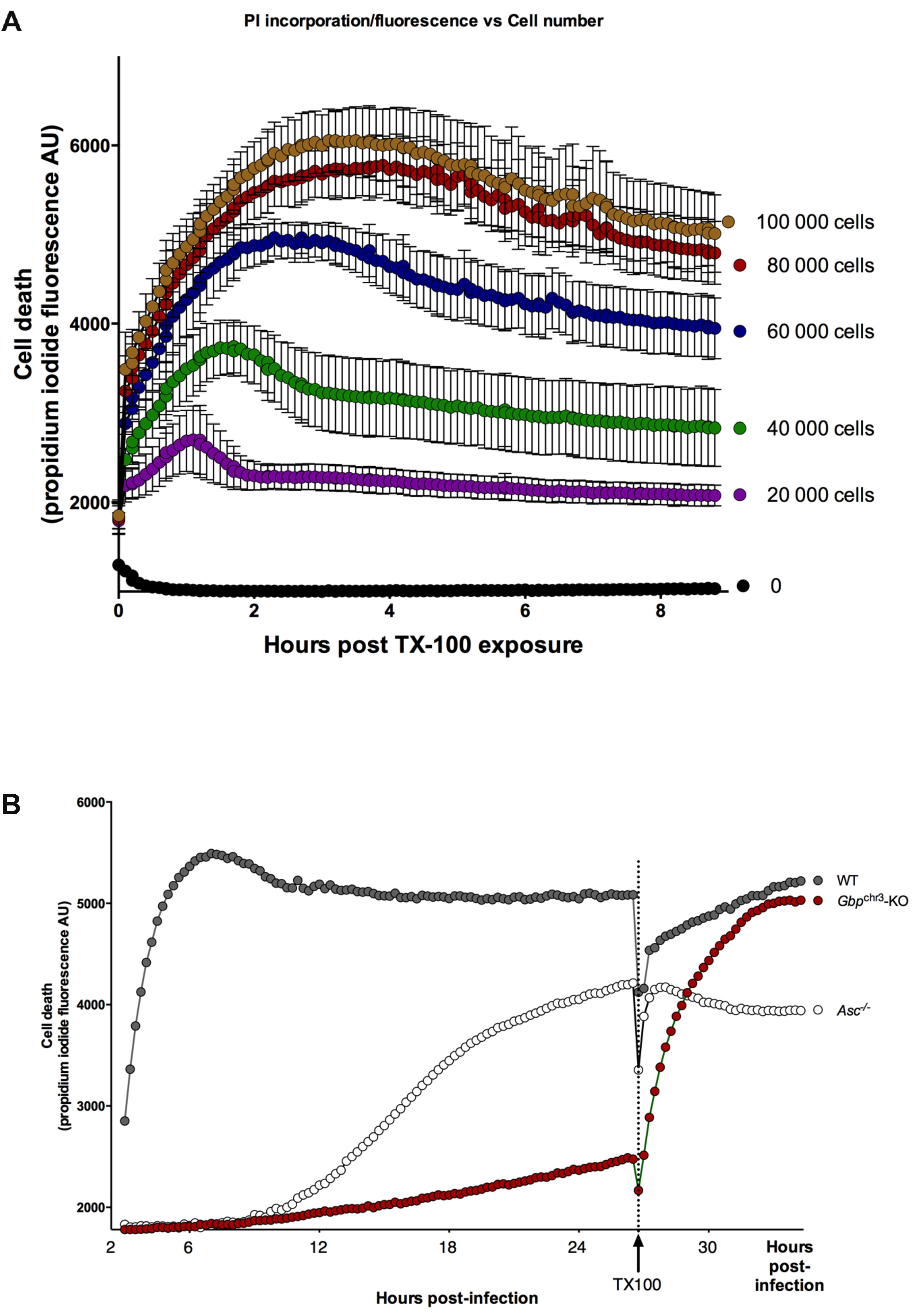

Supplement: S2 Fig — (A) WT BMDMs were seeded at different cell density as indicated and treated with Triton X100 (1% v/v final) in the presence of propidium iodide. Cell death was analyzed in real time by quantifying propidium iodide fluorescence every 5 minutes. (B) IFN-γ-primed BMDMs from the indicated genotypes were infected with F. novicida at a MOI of 10. Cell death was analyzed by quantifying propidium iodide fluorescence every 15 minutes. At 26 h post-infection, TX-100 (1% v/v final) was added leading to a strong increase in fluorescence in Gbpchr3-KO BMDMs but not in WT nor Asc-/- BMDMs confirming that most Gbpchr3-KO BMDMs had an intact (propidium iodide-impermeant) plasma membrane before TX-100 addition in contrast to most WT and Asc-/- BMDMs. One experiment representative of two (A) to at least three (B) independent experiments is shown. (TIF) [file ppat.1006630.s003.tif]

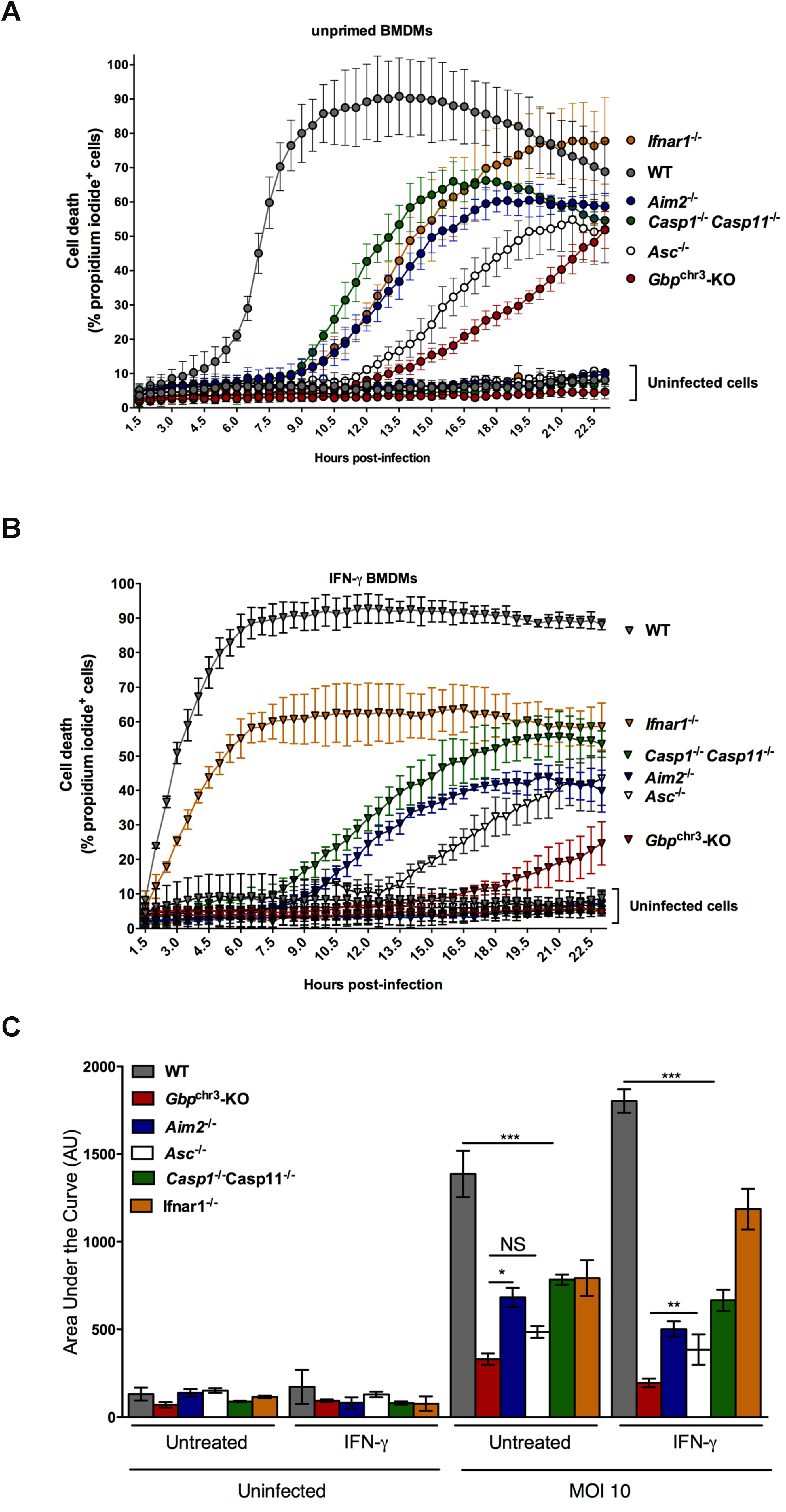

Supplement: S3 Fig — (A,B) Cell death was monitored at single cell level and in real time using automated microscopy and propidium iodide. BMDMs of the indicated genotypes were primed (B) or not (A) with rIFN-γ (100 U/ml) for 16h before infection with F. novicida at an MOI of 10. Automated image analysis was used to quantify the percentage of dead cells at each time points of the kinetics. (C) The area under the curve (corresponding to the above kinetics from 1.5 to 20 h post-infection) was computed. One-way ANOVA analysis was performed with Tukey's correction. (A-C) One experiment representative of two independent experiments is shown. (TIF) [file ppat.1006630.s004.tif]

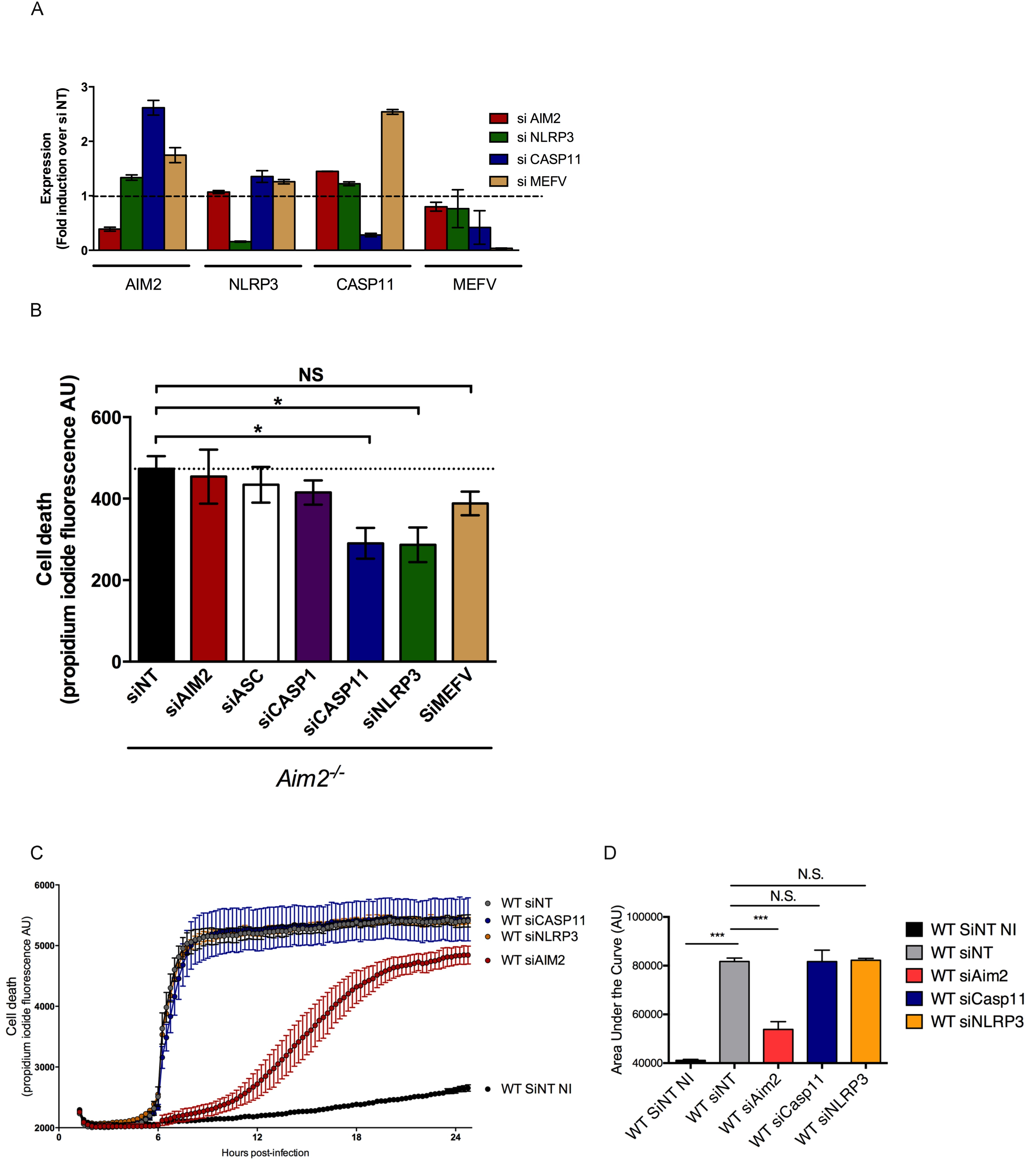

Supplement: S4 Fig — (A) Knock-down efficiency and specificity was determined by qRT-PCR at 48 h post-transfection in BMDMs. The specific transcript levels were normalized to β-actin transcript level and rationalized with the corresponding transcript level in BMDMs treated with a non-targeting (NT) siRNA. (B) Aim2-/- BMDMs transfected with the indicated siRNA were infected with F. novicida at a MOI of 10 and cell death was monitored by measuring propidium iodide fluorescence at 17 h PI. (C) WT BMDMs transfected with the indicated siRNA were infected with F. novicida at a MOI of 10 and cell death was monitored in real time by measuring propidium iodide fluorescence. (D) The area under the curve corresponding to the (C) kinetics from 1 to 20h is shown. NI: Non-infected. (B-D) One-way ANOVA analysis was performed with Tukey's correction. (A-D) One experiment representative of three independent experiments is shown. (TIF) [file ppat.1006630.s005.tif]

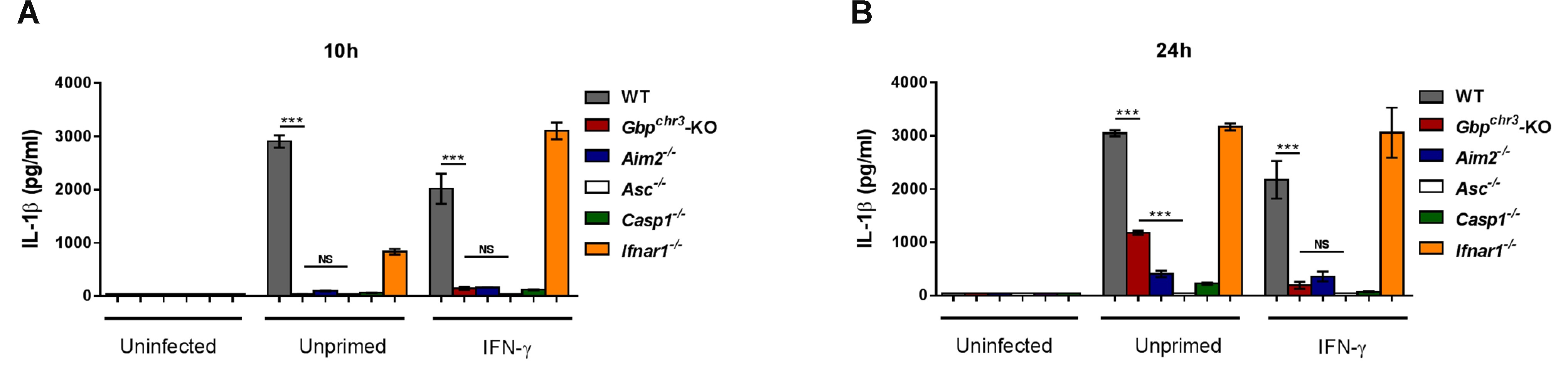

Supplement: S5 Fig — BMDMs from the indicated genotypes were infected or not with F. novicida at a MOI of 10 after priming or not with IFN-γ (100u / ml 16 h). At 10 h post-infection (A) or 24 h post-infection (B) IL-1ß concentrations were determined by ELISA. One experiment representative of three independent experiments with mean and standard deviations is shown. One-way ANOVA analysis was performed with Tukey's correction. (TIF) [file ppat.1006630.s006.tif]

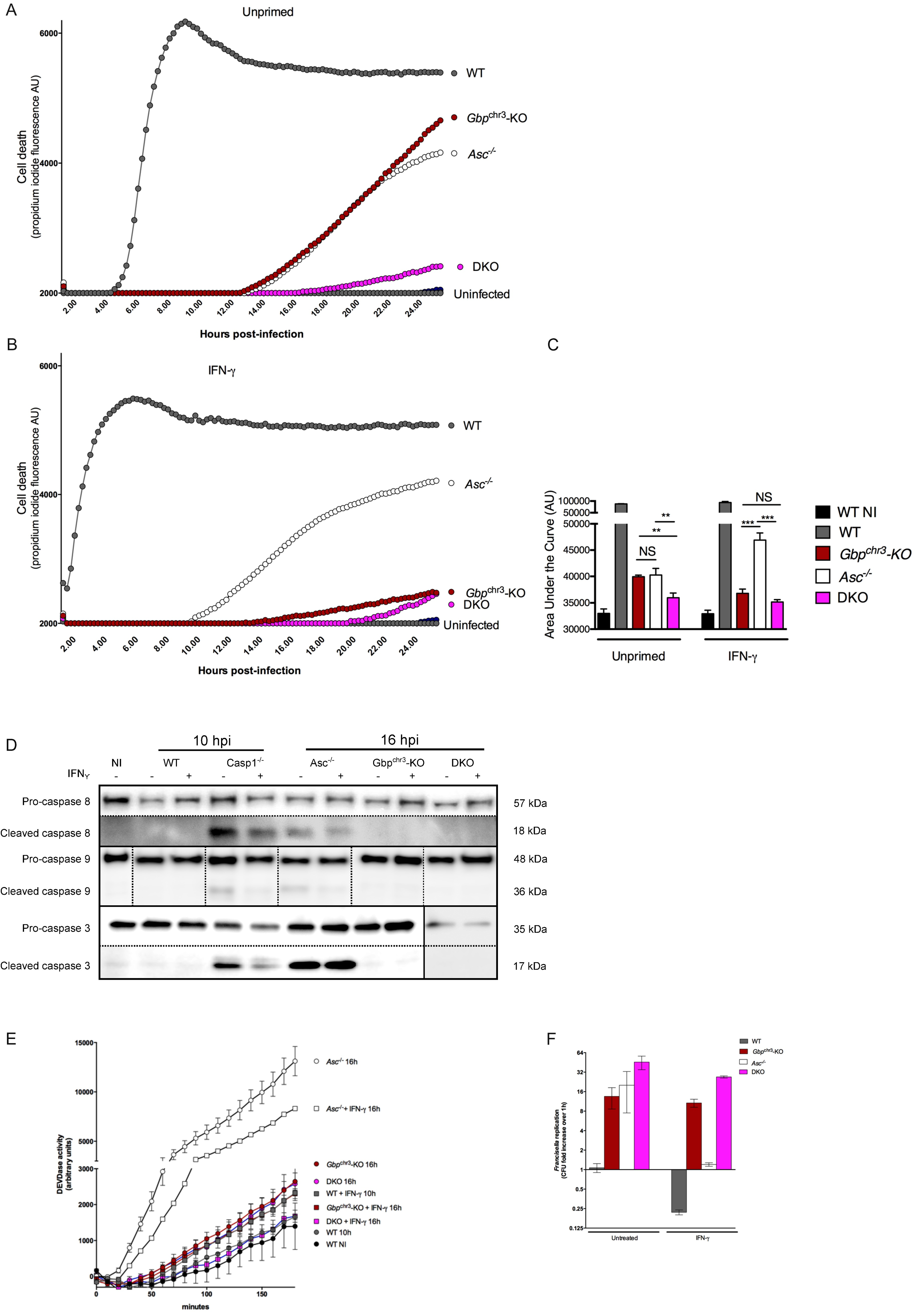

Supplement: S6 Fig — BMDMs from the indicated genotypes (DKO corresponds to Asc-/- GbpChr3-KO doubly-deficient macrophages) were infected or not with F. novicida at a MOI of 10 after priming (B) or not (A) with IFN-γ (100 U/ml, 16 h). (A, B) Real time propidium incorporation/ fluorescence, (C) area under the curve corresponding to the kinetics in A and B, (D) apoptotic caspases processing analysis by Western blotting, (E) DEVDase activity as determined using a fluorogenic caspase-3 substrate and (F) bacterial replication assay by CFU are shown. (C) The dotted vertical lines in Casp9 Western blot illustrate that the samples from a single original Western blot gel/ image were reorganized to fit the indicated order without any other image manipulation. The plain vertical line in Casp-3 Western blot illustrates that the samples from two Western blot gels run and analyzed side by side with the same exposure time are presented. The dotted horizontal lines in Casp8 and Casp3 Western blot indicate images from two different exposure times or from the use of two different primary antibodies (pro- and cleaved Casp3), respectively. The Western blots presented correspond to the ones presented in Fig 4D of the main manuscript. (A, B, C) one experiment representative of two independent experiments is shown. (C) One way ANOVA analysis with Tukey's correction for multiple tests was performed. (D-F) One experiment is shown. Mean and standard deviations are shown. (TIF) [file ppat.1006630.s007.tif]

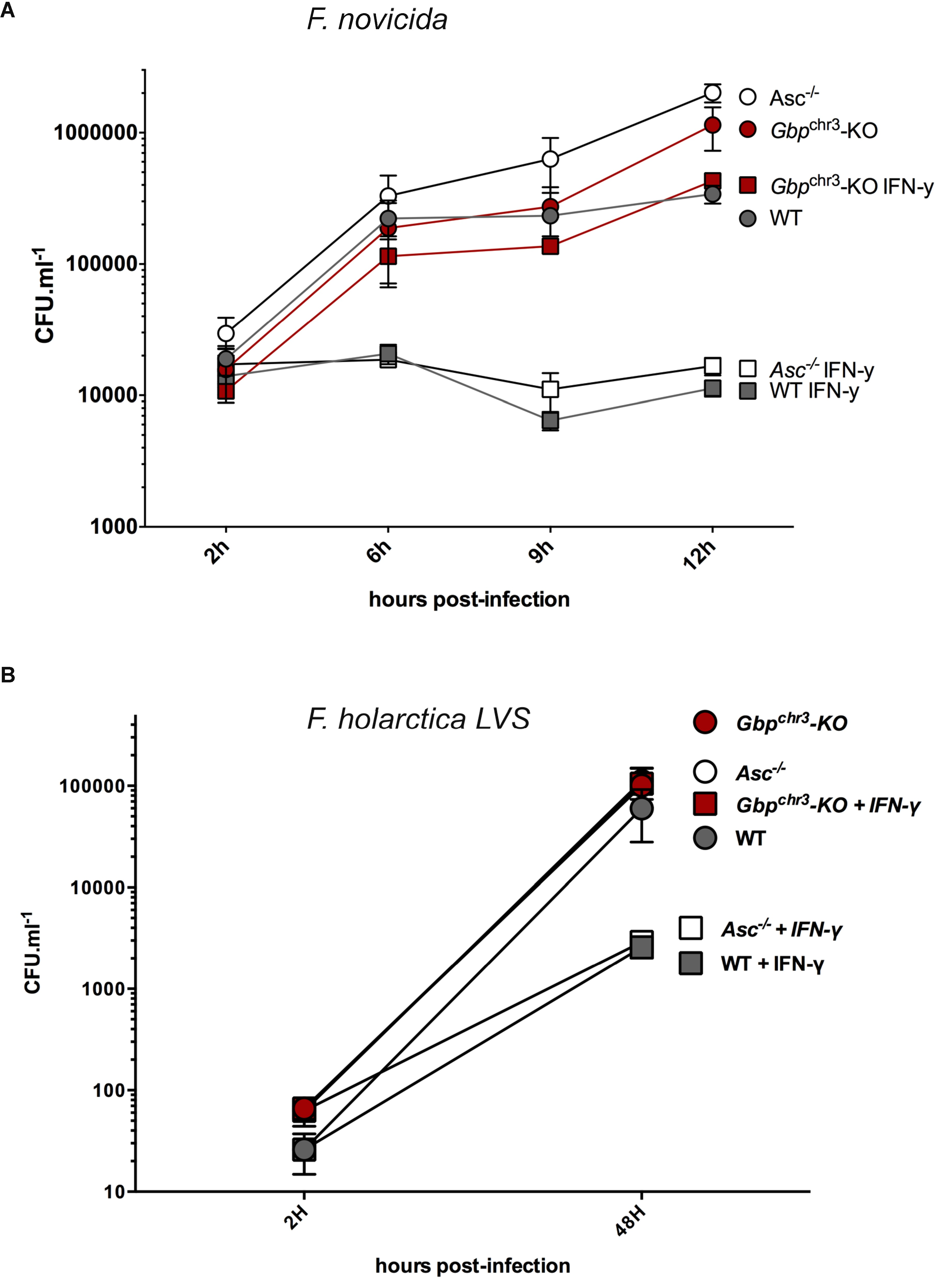

Supplement: S7 Fig — (A-B) BMDMs from the indicated genotypes were primed or not overnight with 100 U/ml of IFN-γ. BMDMs were infected with (A) F. novicida or (B) F. tularensis LVS at a multiplicity of infection (MOI) of 1 and 0.4, respectively. Intracellular bacterial burden was assessed by determination of viable counts at the indicated times post-infection. The corresponding data expressed as Fold increase are presented in Fig 5. (TIF) [file ppat.1006630.s008.tif]

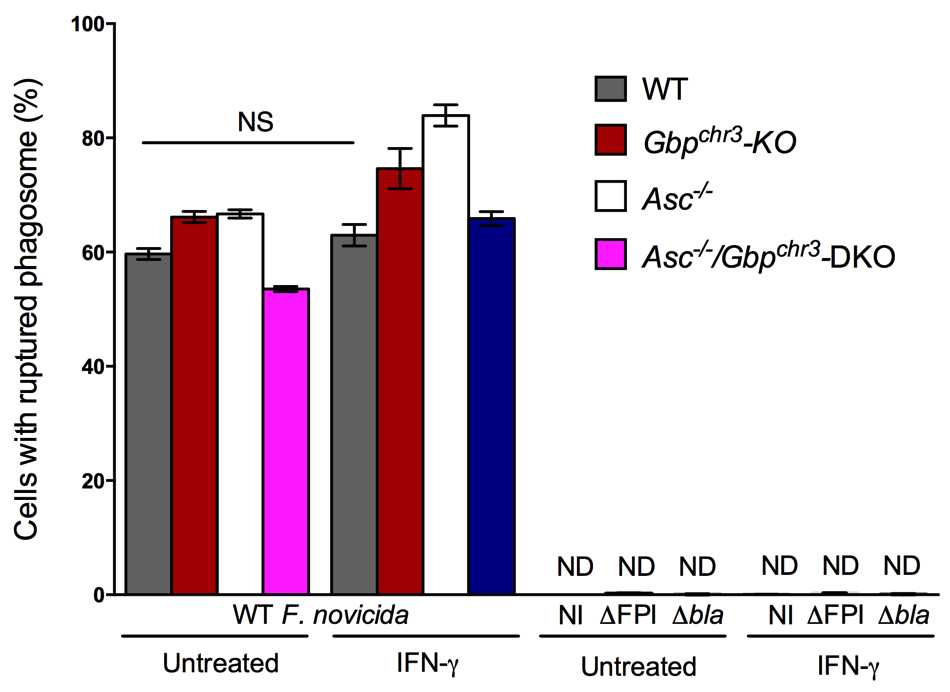

Supplement: S8 Fig — BMDMs from the indicated genotypes were primed or not with 100 U/ml of IFN-γ for 16 h. BMDMs were infected with the indicated F. novicida strains at a multiplicity of infection (MOI) of 10. At 2 h post-infection, cells were incubated with the FRET substrate CCF4. Cytosolic ß-lactamase-mediated CCF4 hydrolysis (a marker of phagosomal permeabilization) was analysed by flow cytometry after gating on live (propidium iodide negative) cells. One experiment representative of two independent experiments is shown. One-way ANOVA analysis was performed with Tukey's correction. (TIFF) [file ppat.1006630.s009.tiff]

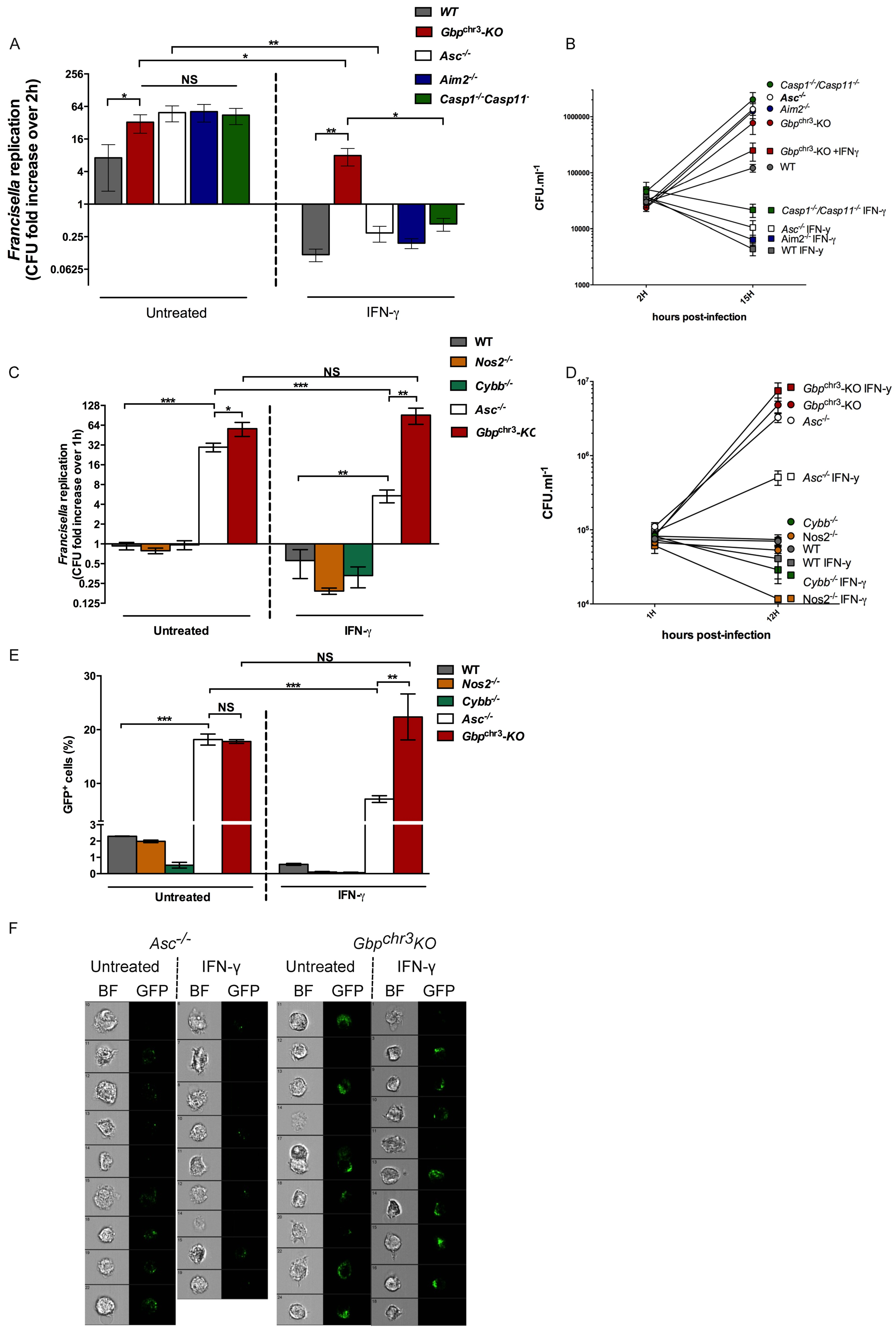

Supplement: S9 Fig — (A-E) BMDMs from the indicated genotypes were primed or not overnight with IFN-γ (100 U/ml) and infected at a MOI of 1 with F. novicida (A-D) or at a MOI of 10 with GFP-expressing F. novicida (E). (A, D) Intracellular bacterial burden was assessed by determination of viable counts at 12 h. (A, C) Results were normalized with the viable counts detected at 2 h post-infection. The corresponding Raw data are presented in (B, D). (E) Flow cytometry-based quantification of infected (GFP+) cells among live BMDMs at 10 h post-infection. (F) Sample ImageStreamX images of BMDMs from the indicated genotypes, treated or not for 16 h with IFN-γ (100 U/ml) and infected at a MOI of 10 with GFP-expressing F. novicida. (TIF) [file ppat.1006630.s010.tif]

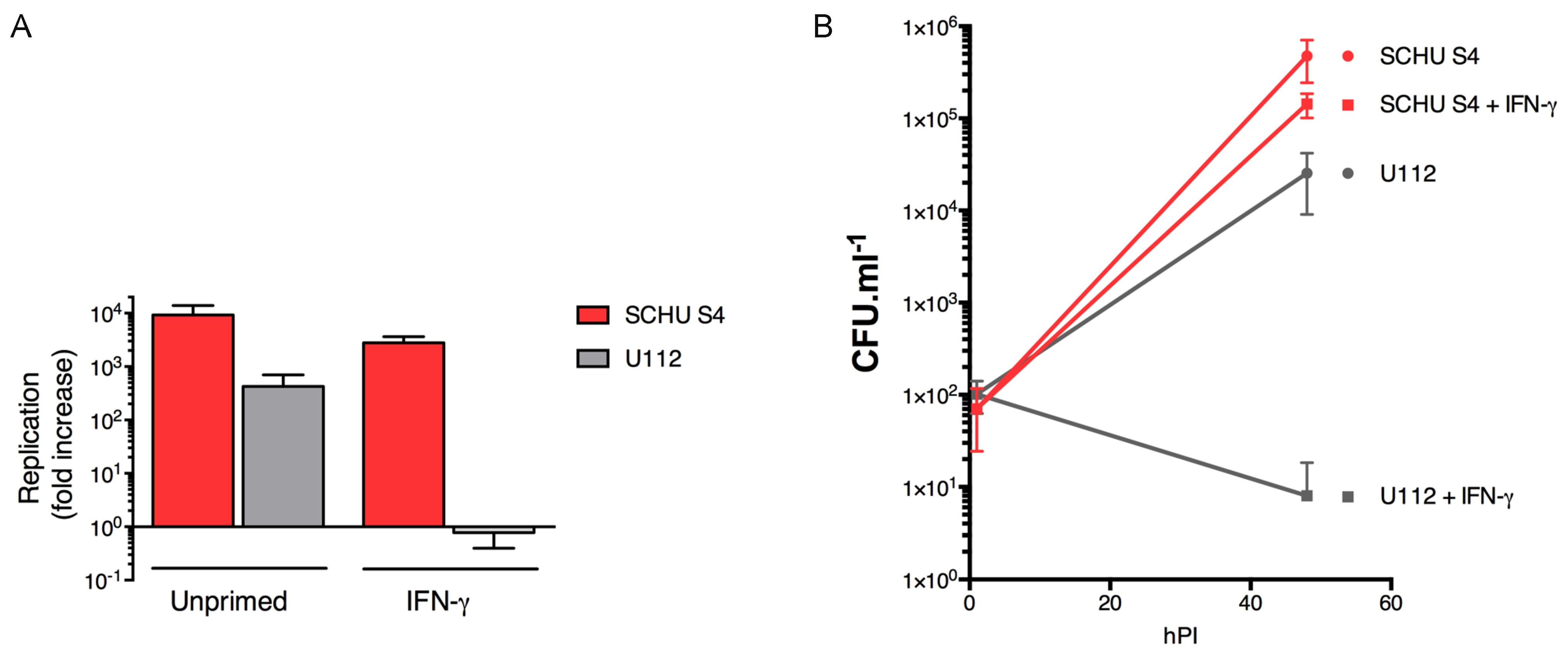

Supplement: S10 Fig — WT BMDMs primed or not overnight with IFN-γ (100 U/ml) were infected at a MOI of 0.4 with F. tularensis SCHU S4 or F. novicida U112. Intracellular bacterial burden was assessed by determination of viable counts at 48 h. (A) Results were normalized with the viable counts detected at 2 h post-infection. (B) The corresponding Raw data are presented. (TIF) [file ppat.1006630.s011.tif]

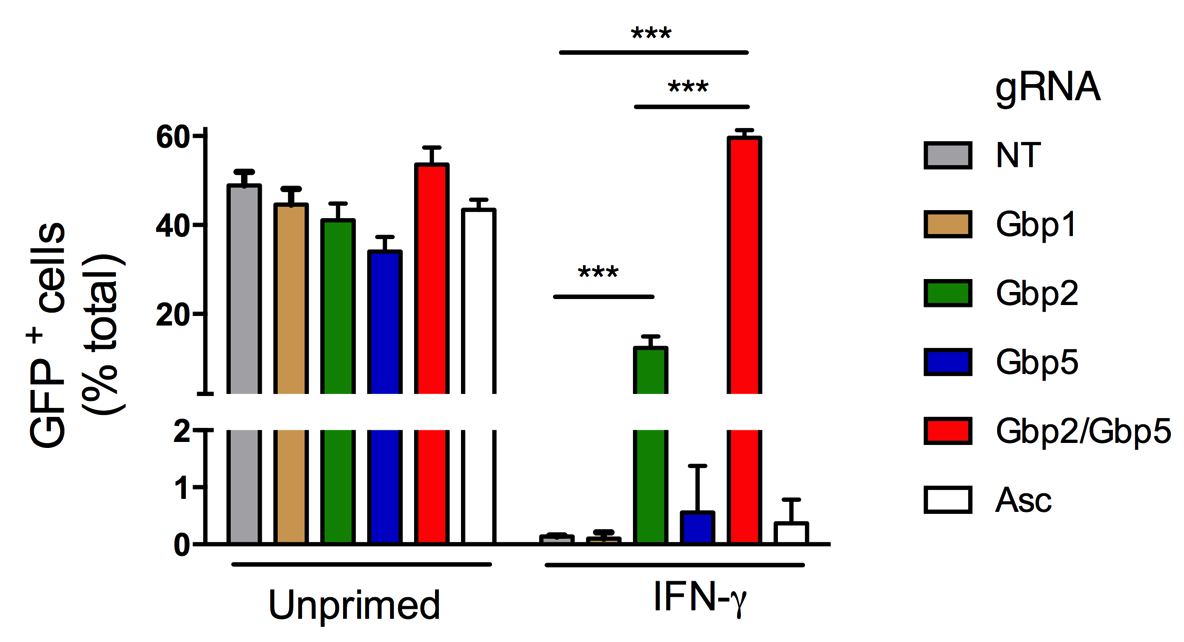

Supplement: S11 Fig — Cas9-expressing J774.1 cells were transduced with non-targeting (NT) gRNA or gRNAs targeting the indicated gene(s). Following puromycin selection, the obtained cell lines primed or not with IFN-γ (100 U/ml for 16 h) were infected with GFP-expressing F. novicida. Live (propidium iodide-negative) cells were analyzed by flow cytometry at 14 h post-infection. One experiment representative of three independent experiments, mean and standard deviations are shown. One-way ANOVA analysis was performed with Tukey's correction. (TIFF) [file ppat.1006630.s012.tiff]

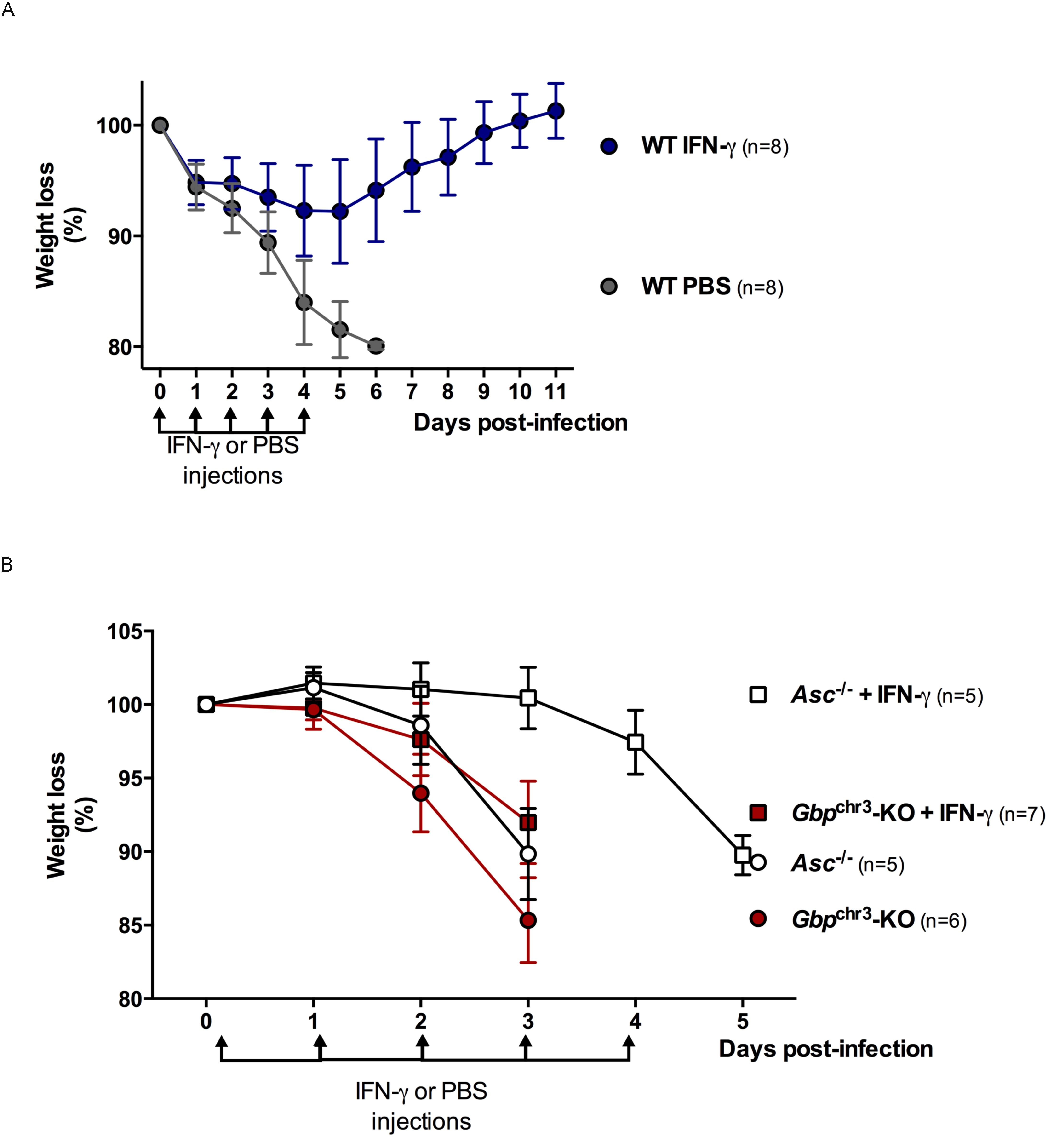

Supplement: S12 Fig — Weight loss of mice of the indicated genotypes (see associated Fig 6) treated by daily i.p injection of PBS or 105 U of rIFN-γ during 5 days after s.c. inoculation with 5×10^4^ (A) or 5x10^3^ (B) F. novicida. (TIF) [file ppat.1006630.s013.tif]

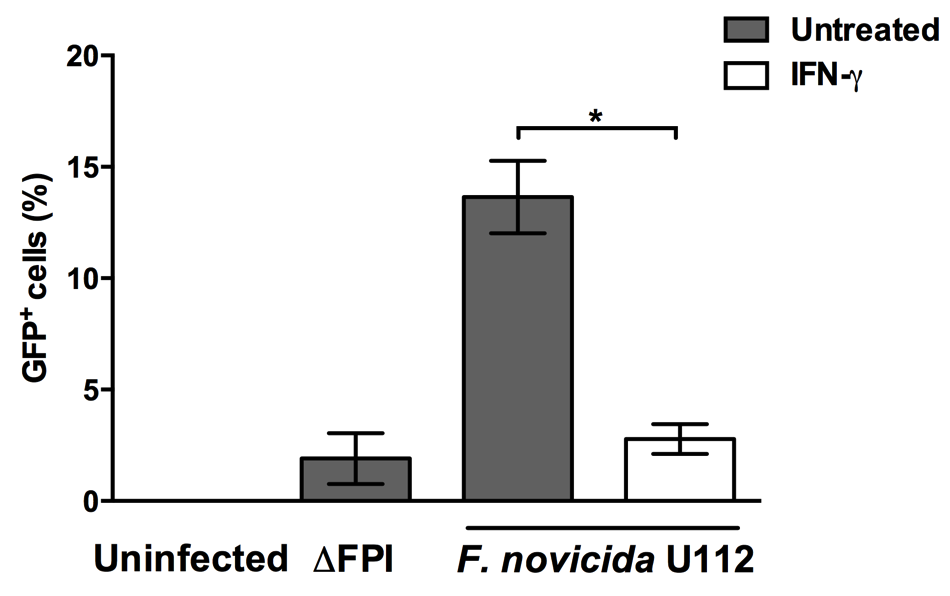

Supplement: S13 Fig — Flow cytometry-based quantification of live infected (PI-, GFP+) primary human macrophages from one healthy donor primed or not with hrIFN-γ (100 U/ml) of and infected for 16 h with GFP-expressing F. novicida strain U112 or the isogenic ΔFPI mutant at a MOI of 1. Mean and s.d. of triplicate wells are shown. Data are representative of two independent experiments. (TIFF) [file ppat.1006630.s014.tiff]
